# Design and self-assembly of hexahedral coordination cages for cascade reactions

**DOI:** 10.1038/s41467-018-06872-0

**Published:** 2018-10-24

**Authors:** Jingjing Jiao, Zijian Li, Zhiwei Qiao, Xu Li, Yan Liu, Jinqiao Dong, Jianwen Jiang, Yong Cui

**Affiliations:** 1 School of Chemistry and Chemical Engineering and State Key Laboratory of Metal Matrix Composites, Shanghai Jiao Tong University, 200240 Shanghai, China; 2 Department of Chemical and Biomolecular Engineering, National University of Singapore, Singapore, 117576 Singapore

## Abstract

The search for supramolecular reactors that contain no catalytically active sites but can promote chemical transformations has received significant attention, but it remains a synthetic challenge. Here we demonstrate a strategy of incorporating bulky and electro-rich aromatic linkers into metallocages to induce cascade reactions. Two hexahedral cages with a framework formula [(Zn_8_**L**_6_)(OTf)_16_] are assembled from six tetrakis-bidentate ligands derived from tetraphenylethylene and eight zinc(II)tris(pyridylimine) centers. The cage cavities can accommodate different molecules such as anthranilamide and aromatic aldehyde through supramolecular interactions, allowing for a cascade condensation and cyclization to produce nonplanar 2,3-dihyroquinazolinones. The reaction is highly efficient with high rate enhancements (up to *k*_cat_/*k*_uncat_ = 38,000) and multiple turnovers compared to the bulk reaction mixture. Control experiments and molecular simulations suggest that the acceleration is attributed to inherent strength of binding affinity for reactants and the release of products to establish catalytic turnover is due to the host−guest geometry discrepancy.

## Abstract (web summary)

Supramolecular containers are promising enzyme mimics, but they currently accommodate only a limited range of chemical transformations. Here, the authors describe coordination cages that catalyze two-component cascade reactions without relying on an external or encapsulated catalytic species.

## Introduction

Self-assembled supramolecular containers have attracted growing attention as enzyme mimetics for potential applications in molecular recognition and storage^1^, sensing^2^, catalysis^3^, and drug transporters^4^. Of particular interest is the possibility of performing chemical transformations in confined spaces, in which the relativities and selectivities may be quite different from those in solution. In light of their well-defined yet tunable structures^5,6^, coordination cages provide an ideal platform for designing supramolecular catalysts for chemical transformations^3^. In this respect, pioneering works have been demonstrated by Fujita^7,8^ and Raymond^9,10^. Some representative reactions include Diels–Alder^11,12^, epoxidation^13^, the aza-Cope rearrangement^14^, Knoevenagel reaction^15^, Nazarov cyclization^16^, sigmatropic rearrangements^17^, and a few others^18–23^. Despite that some examples are known for efficient bimolecular reactions in containers without catalytically active sites, there is a great need for the design of new supramolecular capsules to promote more complicated reactions^7,8,24,25^. Moreover, the range of reaction types is narrow, limiting their use in practical organic synthesis. Cascade or sequential catalytic reactions, as sophisticatedly manipulated by nature, are of great value because such processes can guide the reactive intermediates to the targeted products via consecutive reactions^26,27^. However, it remains unexplored to rationally design catalytic cages for tandem reactions^28,29^. In artificial systems, product inhibition poses another challenge in establishing a catalytic cycle^3^. To address these issues, here we report a strategy of incorporating bulky and electro-rich aromatic linkers into metallocages to induce two-component cascade reactions and reduce product inhibition.

**Fig. 1:**
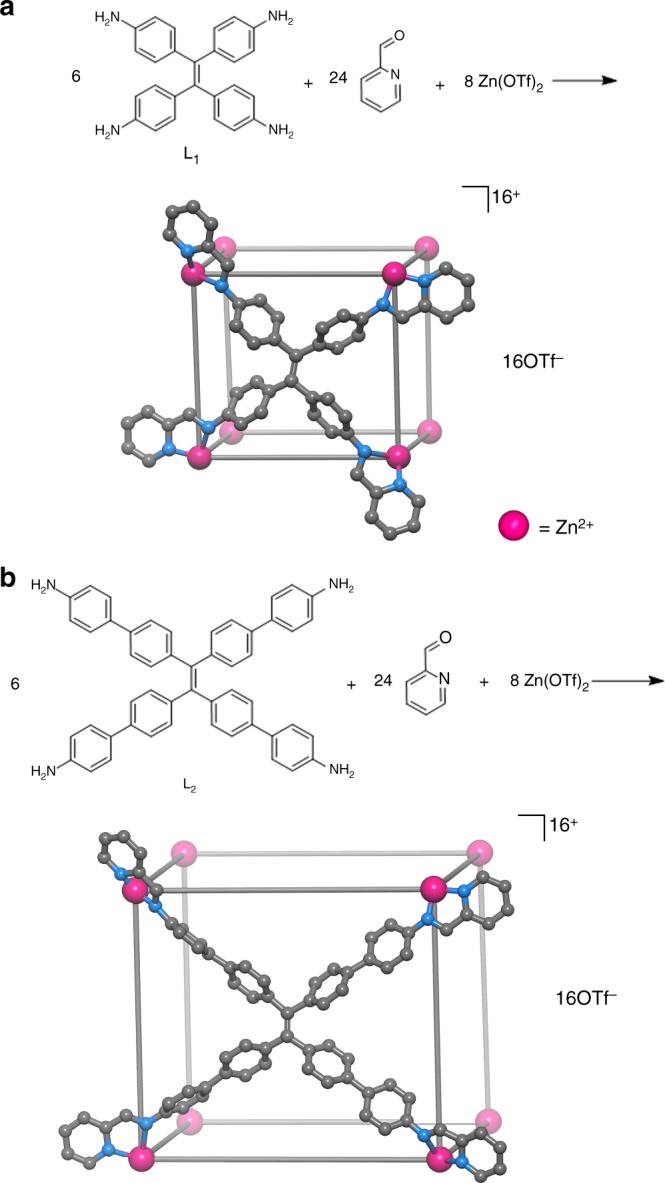
Subcomponent self-assembly. **a** TPE-**1** and **b** TPE-**2**

Coordination cages constructed from organic ligands with extended aromatic panels can provide favorable interactions with aromatic molecules due to high π-electron density of assembled walls^30,31^. We envisioned that specific aromatic−aromatic and/or edge-to-face aromatic interactions may be used for hosts to concentrate aromatic reactants, regulate their orientations and even promote catalytic reactions to generate products that have weak host−guest interactions and can be expelled to allow catalytic turnover^3^. Tetraphenylethylene (TPE) and its derivatives have recently attracted much attention owing to their rich electrochemical and excited state properties^31^ and particularly have been employed for designing cages and metallacycles with aggregation-induced emission^32,33^. Considering their π-electron-rich and structural flexibility, in this study we select two TPE-derived tetraamines as ligands for subcomponent self-assembly of octanuclear Zn_8_L_6_ cages with tunable cavity sizes (Fig. 1). We demonstrate the TPE-based cages could control uptake and release of guests with different shapes and accelerate catalytically the cascade condensation and cyclization of anthranilamide and aromatic aldehydes to nonplanar 2,3-dihyroquinazolinones, with high rate enhancements in comparison to the bulk reaction mixture. The present two cages are rare examples of hollow hosts that can efficiently discern between substrate and product, allowing weak product binding and efficient catalysis.

## Results

### Synthesis and characterization

The ligand **L**_1_ was synthesized according to the reported procedure^34^, and **L**_2_ was synthesized by the Suzuki cross-coupling reaction of (4-nitrophenyl)boronic acid pinacol ester and tetra(4-bromophenyl)ethylene and followed by reduction with Pd/C (Supplementary Figs. 10–12). As shown in Fig. 1, the cages TPE-**1** and TPE-**2** with the general formula [(Zn_8_**L**_6_)(OTf)_16_]·G (G = guest molecule) were obtained by heating Zn(OTf)_2_, **L**_1_ or **L**_2_ and 2-formylpyridine in CH_2_Cl_2_ and CH_3_CN at 70 °C with 64 and 35% yield, respectively. Single crystals of the cages were obtained by diffusion of a mixed solvents of Et_2_O and THF or 1,4-dioxane and THF (1/1, v/v) into a dilute and saturated CH_3_CN solution. The formulations were supported by the results of microanalysis, IR spectroscopy, ^1^H and ^13^C NMR, ^1^H COSY, NOESY, Quadrupole time-of-flight mass spectrometry (Q-TOF-MS), thermogravimetric analyses (TGA) and single-crystal X-ray diffraction.

The ^1^H and ^13^C NMR spectra of each cage displayed only one set of ligand resonances in solution, suggesting the formation of a discrete and highly symmetric assembly (Supplementary Figs. 13 and 14); most of these signals have a slight downfield shift with respect to the free ligand. The *C*_2_-symmetry of the TPE ligand is preserved in the cage, as could be deduced from the number of signals in the NMR spectra. A single peak was observed for each proton of the ligand, with ^1^H-^1^H COSY, NOESY data allowing assignment of each signal (Supplementary Figs. 18 and 19). In diffusion-ordered NMR spectroscopy (^1^H-DOSY) for TPE-**1** (Supplementary Fig. 20a), the observation of a distinct band at log *D* = −9.51 (m^2^ s^−^^1^) with a hydrodynamic radius of about 11.76 Å indicated the formation of single product. Similarly, ^1^H-DOSY of TPE-**2** showed one band at log *D* = − 9.61 (m^2^ s^−^^1^) with a hydrodynamic radius of about 14.79 Å indicating a slightly larger size than TPE-**1** (Supplementary Fig. 20b). Q-TOF-MS analysis provided further support for the existence of multi-tetraphenylethene assemblies, and a clean spectrum was obtained with peaks displaying the expected isotopic patterns at 1083.96, 1331.55, and 1700.41 for TPE-**1** belonging to [Zn_8_(**L**_1_)_6_·*x*CH_3_CN·*y*THF·*z*H_2_O·(16-*n*)OTf]^*n*+^ (*n* = 4−6, Supplementary Fig. 21) and 650.41, 795.89, and 916.64 for TPE-**2** belonging to [Zn_8_(**L**_2_)_6_·*x*dioxane·*y*THF·*z*CH_3_CN·(16-*n*)OTf]^*n*+^ (*n* = 9, 10, 12; Supplementary Fig. 22). TGA revealed that guest molecules in crystalline TPE-**1** and TPE-**2** could be removed in the temperature range from 60 to 160 °C and the materials started to decompose at ~460 °C (Supplementary Fig. 26).

### X-ray crystallography

Single-crystal X-ray diffraction study on TPE**-1** and TPE**-2** revealed the formation of porous hexahedral cages. TPE**-1** crystallizes in the chiral hexagonal space group *P*6_2_22, with one-fourth of the formula unit in the asymmetric unit and three crystallographic *C*_2_-axes passing through three opposite pairs of the ligands. Each of the eight tris(pyridylimine)zinc(II) vertices has an octahedral coordination geometry, with the Zn center chelated by three ligands. The Zn ions in one cage have the same *Δ* or *Λ* configuration. The Zn−N bond lengths range from 2.09 (2) to 2.27(2) Å, which are in good agreement with those reported for related Zn-pyridylimine complexes.

**Fig. 2:**
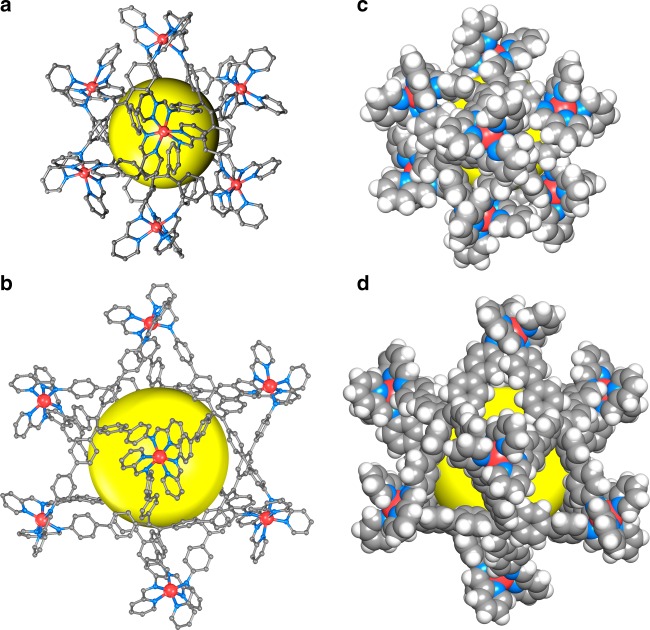
Single-crystal X-ray structures. **a** TPE**-1**, **b** TPE**-2**; **c**, **d** their space-filling models (the cavities are highlighted by yellow spheres). Color coding: Orange, Zn; Blue, N; Gray, C; White, H

Each of the six TPE ligands lying on a two-fold axis coordinates to four zinc atoms through its four chelating pyridylimine groups. The four benzene rings in TPE are non-coplanar, with dihedral angles ranging from 62.9 to 87.6^°^, giving rise to the four-bladed propeller structure in the cage. This arrangement of metal ions and coordination ligands thus leads to a molecular cube with the eight corners occupied by the zinc ions and the six faces by six TPE ligands. The Zn−Zn separations along the cage edges are from 10.27(3) to 11.47(3) Å (Supplementary Fig. 1a) and the ethylene bond separations for TPE between opposite faces are from 10.81(5) to 11.75(11) Å. The cavity of the cage has inner voids around 522.3 Å^3^ (calculated by assuming that the windows are blocked and considering the van der Waals radius) (Fig. 2a, c; Supplementary Fig. 2). The benzene groups of **L**_1_ slightly protrude into the cavity with an irregular window (3.7 × 7.8 Å^2^), generating portals to allow the ingress/egress of guests (Supplementary Fig. 1b).

The large cage TPE**-2** crystallizes in space group $P\overline 1$, with the whole formula unit in the asymmetric unit. Due to the increased rotational freedom from **L**_1_ in TPE**-1** to **L**_2_ in TPE**-2**, the *C*_2_ symmetry was broken. TPE**-2** has a similar hexahedral structure assembled from eight zinc ions and six **L**_2_ ligands. The Zn−Zn distances along the edges of the hexahedron range from 14.59(5) to 18.34(7) Å (Supplementary Fig. 3a), and the ethylene bond separations between the opposite faces are in the range of 17.10(3)−18.45(3) Å. Space-filling representations of TPE**-2** clearly show the formation of a large porous cage with a volume of about 2222.4 Å^3^ (Fig. 2b, d; Supplementary Fig. 4), and the longer ligands **L**_2_ reduce an irregular wide window (13.7 Å × 6.4 Å^2^) (Supplementary Fig. 3b). So, TPE**-2** offers a much larger and flexible cavity than TPE**-1**. Notably, in both cases, the C−H bonds in the highly twisted aromatic rings are oriented toward the inside of cavities, offering potential for strong supramolecular interactions and the encapsulation of guest molecules. This is different from most of the rigid coordination cages for host−guest chemistry^7,9,21^.

### Supramolecular catalysis

The present porous hexahedral cages featuring rich π-electron density may accommodate aromatic guests, which is beneficial for chemical transformation and catalysis in the cavities. We therefore employed the two cages for supramolecular catalysis by taking advantage of their hydrophobic and flexible cavities with tunable size. In this study, we focus on the catalytic synthesis of 2,3-dihydroquinazolinones, which are an important class of fused heterocycles due to their pharmacological activities, such as antitumor, analgesic, antifibrillatory, antibiotic, antispermatogenic, and vasodilatory efficacy^35^. The catalytic activities of the cages were evaluated by using anthranilamide (**3a**) and 4-fluorobenzaldehyde (**4b**) as substrates. After screening various reaction conditions including catalyst loading, reaction temperature, and solvent (Supplementary Table 8), we found that, in the presence of 0.1 mol% of TPE**-1**, the reaction of anthranilamide and 4-fluorobenzaldehyde proceeded smoothly in a mixture of CH_3_CN and toluene at 40 °C. Notably, a lower catalyst loading (0.05 mol%) can be used, but prolonged reaction time (45 h) was required. We measured the reaction rate under this condition by ^1^H NMR spectroscopy: the starting material transformed into intermediate **5b** and product **6b** nearly completely in 3 h and completed within 11 h to furnish the targeted 2,3-dihydroquinazolinone (**6b**) in 95% yield. Besides, the reactions of anthranilamide with other aromatic aldehydes such as benzaldehyde, 4-methylbenzaldehyde, and 4-methoxybenz aldehyde can also be catalyzed by TPE-**1**, which afforded 76−81% yields of the products (Table 1). Anthranilamide with electron-donating or -withdrawing substituents (−Me or −Cl) on the phenyl group afford the targeted products with excellent yields (entries 19 and 21, Table 1).

**Table 1:** Sequential condensation and cyclization of anthranilamide with aldehydes catalyzed by the cages (for reaction details, see Experimental section)


Entry	Catalyst	Catalyst loading^a^ (mol%)	*R*_1_	*R*_2_	Yield (%)^b^
1	TPE-**1**	0.1	H	Ph	**6a**/81
2	TPE-**2**	0.1	H	Ph	**6a**/90
3	TPE-**1**	0.1	H	4-FPh	**6b**/95
4	TPE-**2**	0.1	H	4-FPh	**6b**/99
5	TPE-**1**	0	H	4-FPh	**6b**/0
6	TPE-**2**	0	H	4-FPh	**6b**/0
7	**L**_1_ or **L**_2_	0.6	H	4-FPh	**6b**/0
8	2-PyCHO^c^	2.4	H	4-FPh	**6b**/0
9	Zn(PI)_3_^d^	0.8	H	4-FPh	**6b**/0
10	Bu_4_NOTf	1.6	H	4-FPh	**6b**/0
11	TPE-**1**	0.1	H	4-MeOPh	**6c**/78
12	TPE-**2**	0.1	H	4-MeOPh	**6c**/87
13	TPE-**1**	0.1	H	4-MePh	**6d**/76
14	TPE-**2**	0.1	H	4-MePh	**6d**/92
15	TPE-**1**	0.1	H	1-naphthyl	**6e**/0
16	TPE-**2**	0.1	H	1-naphthyl	**6e**/99
17	TPE-**1**	0.1	H	9-anthral	**6f**/0
18	TPE-**2**	0.1	H	9-anthral	**6f**/0
19	TPE-**1**	0.1	Me	4-FPh	**6g**/99
20	TPE-**2**	0.1	Me	4-FPh	**6g**/99
21	TPE-**1**	0.1	Cl	4-FPh	**6h**/94
22	TPE-**2**	0.1	Cl	4-FPh	**6h**/96

^a^Catalyst loading based on anthranilamide

^b^Isolated yield

^c^2-PyCHO = 2-formylpyridine

^d^Zn(PI)_3_ = tris(pyridylimine)zinc(II)bis(triflinate)

**Fig. 3:**
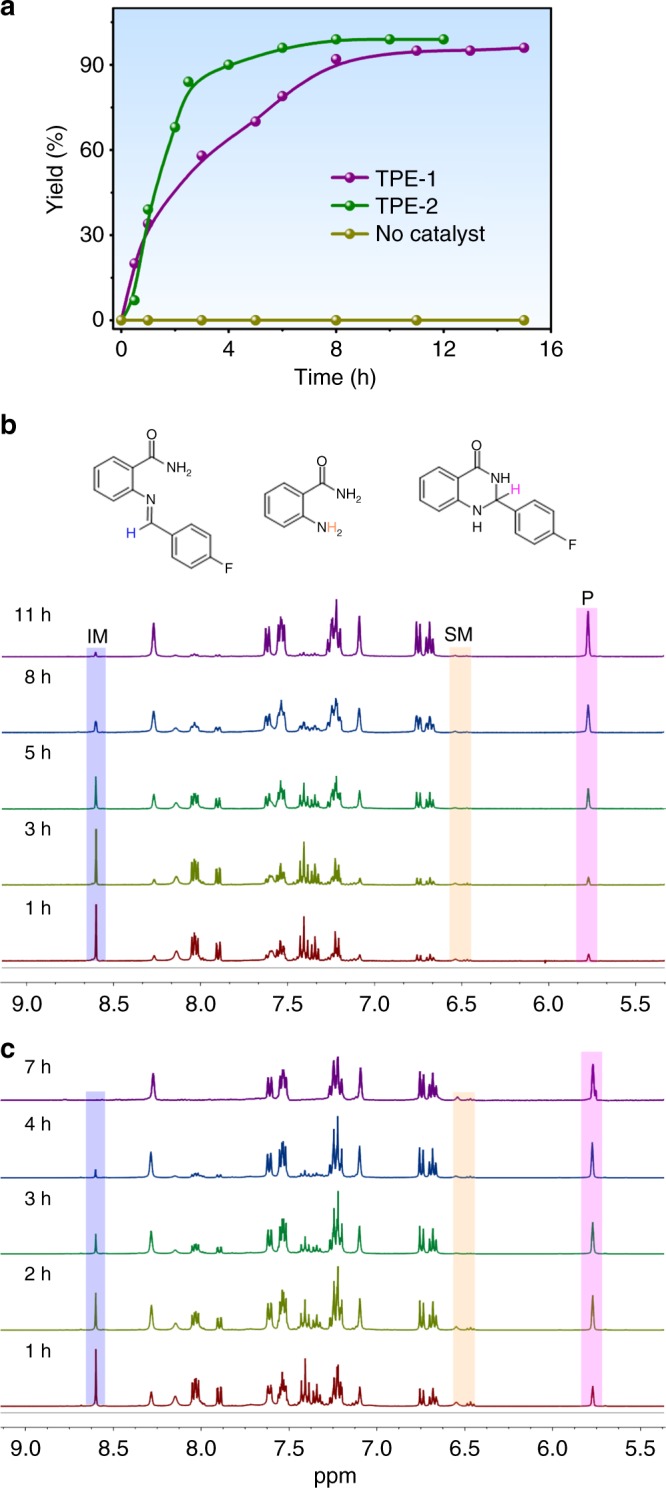
Kinetic results of the catalysis. **a** Kinetic curves obtained with 0.1 mol% of the cage and without catalyst in the sequential condensation and cyclization of **3a** and **4b**; **b**, **c** evolution of ^1^H NMR spectra during the reaction for TPE**-1** and TPE**-2** [SM = starting material (**3a**); IM = intermediate (**5b**); *P* = product (**6b**)]

Under identical conditions, TPE-**2** is found to be a more efficient catalyst than TPE-**1** for the above sequential reactions (Fig. 3); probably due to that it has a much larger pore for substrate exchange. Specifically, the reactions catalyzed by 0.1 mol% loading of TPE**-2** were complete within 7 h monitored by ^1^H NMR spectroscopy (Fig. 3b, c), affording 87−99% isolated yields. A ^1^H NMR study of the precipitate, which was recovered from the reaction mixture by adding ethyl ether, did not show any trace of the encapsulated product, signifying the weak interaction of the product with the cage cavity. It was apparent that sequential reactions of anthranilamide and aldehyde were accelerated by the cages. To measure the rate constant in the presence of cage (*k*_cat_) with little interference from the background reaction (*k*_uncat_), we assessed the model of Michaelis−Menten scheme (Supplementary Figs. 29 and 30)^21^. As expected, kinetics achieved saturation at high substrate concentrations and showed a first dependence on the catalyst loading (Supplementary Fig. 31). The *k*_cat_/*k*_uncat_ ratios were found to be 1.1×10^4^ and 3.8×10^4^ for TPE**-1** and TPE**-2**, respectively. Therefore, the cages reported here can be potentially acted as functional enzyme mimic in catalysis^21,36^. The catalytic activities observed for the two cages are comparable well with those reported for strong Lewis acids such as Zn(OTf)_2_, Sc(OTf)_3_^37^ and Ga(OTf)_3_^38^ or strong Brønsted acids such as sulfonic acid^35^ and phosphoric acid^39^ at 1.0 mol% catalyst loading (Supplementary Fig. 33). When the loading reduce to 0.1 mol%, all of these catalytic systems failed to promote the cyclization and imine was observed as a major product. The rate accelerations of the catalyzed reaction over the uncatalyzed reaction are on the order of 10^4^, which are well comparable with those reported for supramolecular catalysis in metal-ligand hosts (Supplementary Table 5)^9,12^.

To prove that the efficient catalysis occurs predominantly within the cage cavities, we conducted a series of extensive experimental studies. First, we added an excess of a strongly binding competing guest (2.5 mmol pyrene, *K*_a_ = 2.8×10^5^ M^−^^1^ and 2.0×10^5^ M^−^^1^ for TPE**-1** and TPE**-2**, respectively) (Supplementary Fig. 44). With this inhibitor present, the reaction cannot proceed at all because the competing guest prevented substrate binding in the cage cavity. Second, control experiments showed that, in the absence of the cages, the reaction of **3a** and **4b** only gave the imine intermediate about 47% yield at 40 °C after 14 h (Table 1, entries 5 and 6; Supplementary Fig. 28). Meanwhile, when 2-formylpyridine (2.4, 240, and 24,000 mol%), Bu_4_NOTf (1.6 mol%), the ligand **L**_1_ (or **L**_2_, 0.6 mol%) or a mononuclear tris(pyridylimine)zinc(II) complex (0.8 mol%) (Supplementary Figs. 5, 15 and 23), prepared from 2-formylpyridine and *p*-toluidine was used, no sequential reaction was observed in each case (Table 1, entries 7−10). Furthermore, inductively coupled plasma emission spectrometry (ICP-AES) of the aqueous phase after extracting the reaction mixture indicated 0.01 and 0.02% loss of Zn ions for TPE-**1** and TPE-**2**, respectively, which excludes the catalytic effect of the free Zn ions. Third, competitive size selectivity studies were also performed. When 1-formylnaphthalene that contains two aromatic rings was employed, TPE**-2** efficiently catalyzed the reaction (95% yield), but TPE**-1** cannot promote the reaction at all (Table 1, entries 15 and 16). However, when sterically bulky 9-anthracenealdehyde bearing three aromatic rings was employed (Supplementary Fig. 9), both TPE**-1** and TPE**-2** cannot promote the reaction, probably due to that the bulky substrate cannot enter into the cavities through the windows (Table 1, entries 17 and 18). Both TPE-**1** and TPE-**2** have an irregular window with dimensions about 3.7 × 7.8 Å^2^ and 6.4 × 13.7 Å^2^, respectively. Despite the well-defined structure, the host ligand framework and the cage cavity are flexible, especially in which the aromatic rings can rotate to adjust portal sizes, and so the substrates such as **3a** (5.8 × 6.0 Å^2^) and **4b** (6.0 × 8.5 Å^2^) with sizes smaller and even slightly larger than the portals can enter the cavities from windows^40,41^. However, the substrates are too large, so they cannot enter the cage cavities [1-formylnaphthalene (8.0 × 8.5 Å^2^) vs. TPE-**1** and 9-anthracenealdehyde (8.0 × 10.9 Å^2^) vs. TPE-**1** and TPE-**2**]. Taking together, the above results suggested that the sequential condensation and cyclization reaction was indeed associated with the substrates being bound in the cage cavity. We demonstrated that the catalytic reaction occurs with a lot of turnovers. To a solution of TPE**-1** (0.1 mol% loading) in CH_3_CN and toluene, we added several successive portions of anthranilamide and 4-fluorobenzaldehyde, waiting until each aliquot had completely reacted before adding the next. We can see from Supplementary Fig. 32 that, after multiple additions of reactants, the reaction profile remained unchanged, and so there is no detectable change in activity after five and even ten turnovers. Moreover, after conversion of all substrates to the product, the cages were recovered and found to retain the structure intact, as evidenced by ^1^H NMR, TOF mass, and UV−Vis spectra (Supplementary Figs. 16, 17, 24, 25 and 27).

### Host−guest interactions

To further understand the host−guest interactions, we studied the ability of the cages to encapsulate reactants and products by ^1^H and ^19^F NMR, IR and UV–Vis titration. First, in the presence of the reactants **3a** and **4b**, we observed the downfield shifts of ^1^H NMR proton signals of reactants (Supplementary Figs. 34 and 35). For example, in a solution of TPE**-1** and **3a** or **4b**, a 0.17 ppm downfield shift was observed for the proton on the aromatic ring of **3a** at 6.52 ppm and 0.04 ppm downfield shift for the proton on **4b** at 6.36 ppm (Supplementary Fig. 34), and in a solution of TPE**-2** and **3a** or **4b**, a 0.10 ppm downfield shift was observed for the proton on the aromatic ring of **3a** at 6.69 ppm and 0.03 ppm downfield shift for the proton on **4b** at 7.40 ppm (Supplementary Fig. 35). The downfield shift of ^19^F peak of **4b** at 104.61 ppm in the presence of TPE-**1**/TPE-**2** to 78.66 ppm indicates the encapsulation behavior (Supplementary Fig. 36). The host–guest interactions were further confirmed by ^1^H-DOSY in 50:50 (v/v) DMSO-*d*_6_/CD_3_CN, in which the diffusion coefficients were single set of resonances in the presence of substrates (Supplementary Figs. 37 and 38). For the mixture system of **3a** + **4b**, both the same value of diffusion coefficient for the substrate mixture and TPE-**1**/TPE-**2** and crossover signals for protons of **3a**/**4b** with protons on the benzene rings of TPE-**1**/TPE-**2** indicated simultaneous encapsulations of the two substrates in the cage (Supplementary Figs. 37-39). It should be noted that guest molecules trapped by a host cavity typically exhibit upfield shifts in ^1^H NMR, although trapped molecules that display upfield shifts were also observed^25,42^. In this work, the cages were constructed with TPE units as faces that contribute a great electron conjugate system around the periphery of cages. When guest molecules moved into the cavity, they may be in the de-shielding distinction of benzene rings. Besides, the formation of favorable CH···π, CH···N and CH···O interactions upon encapsulation also provided a plausible structural rationale for the downfield shifts.

**Fig. 4:**
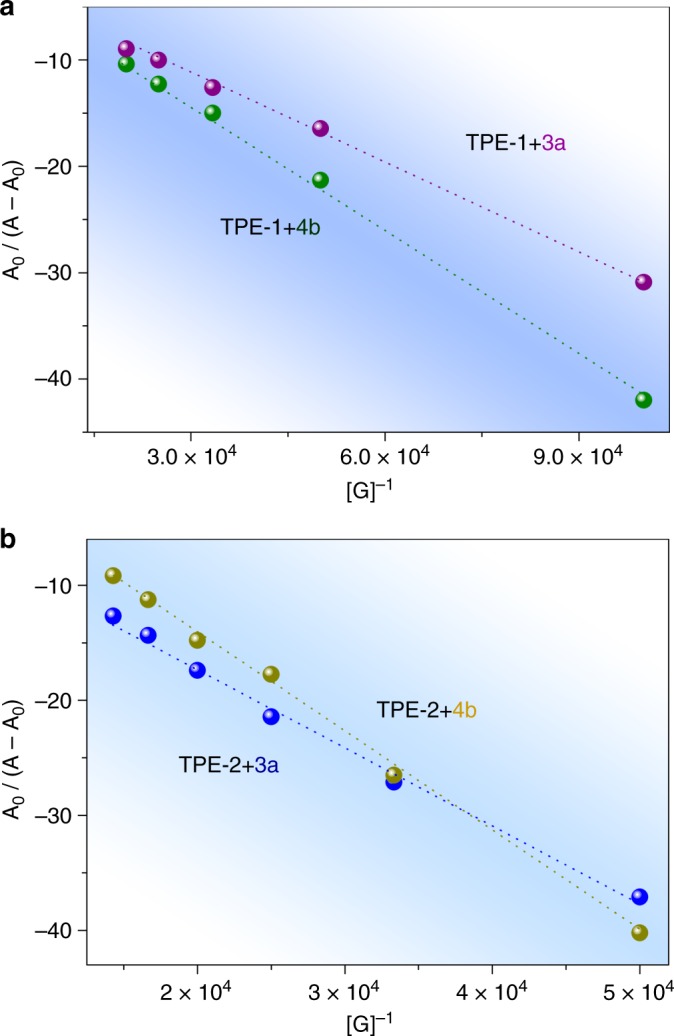
Benesi−Hildebrand plots. **a** TPE**-1** titration with **3a** and **4b**; **b** TPE**-2** titration with **3a** and **4b**. The plots were obtained by using a UV−Vis titration experimental method in CH_3_CN at r.t

UV−Vis titration experiments were performed to study the guest binding ability of the cages (Supplementary Figs. 40 and 41). As shown in Fig. 4, a better fit was obtained to a 1:1 host−guest isotherm, and the associate constants (*K*_a_) were found to be 4.0×10^4^ and 1.2×10^4^ M^−^^1^ for TPE**-1** and the analytes **3a** and **4b**, respectively, and 1.5×10^4^ and 1.0×10^4^ M^−^^1^ for TPE**-2** and **3a** and **4b**, which are much higher than the *K*_a_ values of 1191 and 1754 M^−1^ observed for the two cages and the intermediate **5b**, and 874 and 215 M^−1^ observed for the two cages and the product **6b** (Supplementary Figs. 42 and 43), respectively. The quite different *K*_a_ values suggested that the cages catalyzed the reaction as a turnover process based on uptake of the substrate and release of the product. The formation of host−guest adducts was also revealed by solid-state IR spectra, which showed the characteristic peaks of υ(N−H) at ~3440 cm^−1^ and υ(C=O) at ~1697 cm^−1^ for **3a/4b**@TPE-**1**/**2** (Supplementary Figs. 45 and 46). While no such characteristic stretching bands due to **6b** were detected (Supplementary Fig. 47), indicating that the product was not adsorbed in the cages.

### Molecular dynamic simulation

In order to provide microscopic insight into the cavity-confined effect, molecular dynamics (MD) simulations with the universal force field^44^ were conducted to study the origin of host−guest complexes and estimate their interaction energies under no solvent environment. As shown in Supplementary Fig. 6, both the substrates **3a** and **4b** can be trapped in the cavity of TPE**-1** or TPE**-2**. Each of them is involved with several host−guest interactions including CH···π interactions (2.68-3.32 Å) and hydrophobic CH···N and CH···O interactions (2.35-3.78 Å). Importantly, the cavity-confined microenvironment triggered the inherent strength of binding affinity between the two reactants via π···π and hydrophobic interactions (2.65~3.62 Å). For the substrates **3a** + **4b**, **3a** + **4e** and **3a** + **4f** in TPE**-1**, the binding energies were calculated to be −34.98, 44.39, and 52.67 kcal mol^−1^, respectively (Supplementary Fig. 7 and Supplementary Table 7). The negative value of −34.98 for **3a** + **4b** suggests that the confinement in TPE-**1** is favorable for host−guest interaction and subsequent condensation reaction. With increasing molecular size of **4**, the energies in TPE-**1** becomes positive, which implies that the cage is not sufficiently large to accommodate **3a** + **4e** or **3a** + **4f**, thereby leading to the steric hindrance inhibition of the reaction. Unlike TPE**-1**, the energies are found to be −126.95, −94.65, and 18.65 kcal mol^−1^ for **3a** + **4b**, **3a** + **4e** and **3a** + **4f** in TPE**-2**, respectively. Obviously, TPE**-2** has a larger cavity to accommodate **3a** + **4e** with stable interaction and thereby can promote the reaction (in addition to **3a** + **4b**). Nevertheless, the bulky reactants **3a** + **4f** cannot fit into the cavity of TPE**-2** and their condensation reaction is impeded.

It should be noted that the binding energies from simulations were based on an implicit solvent model. If the solvent molecules were included, the simulations would be quite time-consuming. Therefore, there is a large difference in the binding energies of **3a** + **4b** with TPE-**1** and TPE-**2**. In experiments, the solvent was present and, to a large extent, screened the binding, thus leading to a small difference in the associate constants obtained from UV−Vis titration. Nevertheless, these calculated interaction energies are consistent with the experimental observations of cavity-confined effect in the two cages. We believe that rate acceleration of this reaction is associated with the enhanced binding of the reactants in the confined cavity.

### Proposed catalytic mechanism

**Fig. 5:**
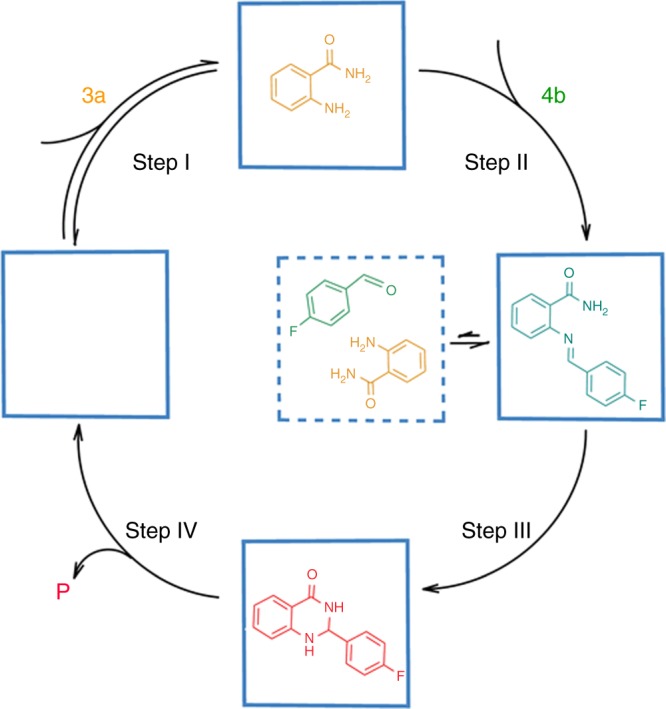
Representation of the sequential condensation and cyclization of anthranilamide with aldehyde in the cage (the square represents the cage; **3a** and **4b** stand for the substrates and **P** stands for the product)

Based on the above experimental studies and molecular simulations, we propose the following reaction mechanism (Fig. 5) and attribute the cage cavities to co-encapsulate the two different reactants to promote the reaction. First, according to the UV−Vis titration results, both cages prefer to accommodate anthranilamide **3a** (higher *K*_a_ than reactant **4b**) within their hydrophobic cavities (Step I). Second, the reactant **4b** enter cavity and stack with encapsulated **3a** face to face in the most stable configuration. The model of the host−guest binding system was conducted by molecular dynamic simulations revealing the enough negative binding energy (Supplementary Fig. 6). This special binding event in the cavity may reduce the reaction energy barrier, leading to transformation of the intermediate **5b** immediately (Step II). Third, the reaction of a subsequent intramolecular nucleophilic attack of the amide nitrogen on the activated imine group is followed by a 1,5-proton transfer to yield the final product in cages (Step III). The rich electronic and conjugate TPE faces of the cages may facilitate the deprotonating of the amide nitrogen via supramolecular interactions to render it more nucleophilic in the cyclization^43^. This is similar to the same cyclization reaction catalyzed by Lewis acids^40^ or Brønsted acids^37^ (Supplementary Fig. 33). However, further study is greatly needed to understand the tandem reaction mechanism with a coordination cage. Fourth, the sequential reaction thus breaks the planarity of aromatic substrates (Supplementary Fig. 8), promoting dissociation of product to allow catalytic turnover (Step IV). The weak product binding was evidenced by the much smaller associate constants with cages than reactants **3a** and **4b** in UV−Vis titrations (Supplementary Figs. 40 and 41). Besides, we did not observe the reaction rate decreasing with time, which means that the reaction cannot be inhibited by accumulation of product.

## Discussion

We have described the design and preparation of two TPE-faced hexahedral Zn_8_**L**_6_ coordination cages with tunable cavity sizes by subcomponent self-assembly. The flexible and hydrophobic cavities of the two cages favor inclusion of aromatic substrates via CH···π and hydrophobic interactions and can efficiently encapsulate anthranilamides and aromatic aldehydes and accelerate catalytically a sequential condensation/amine addition of the guests to bent-shape 2,3-dihyroquinazolinones, with a maximum observed rate acceleration of 38,000-fold. Because of their unfavorable nonplanar configurations, the products can be easily expelled from the cage cavities to allow multiple catalytic turnovers. The host–guest investigations between the cages and reactants/products have been examined by a variety of spectroscopic techniques and molecular dynamics simulations. Manipulation of steric and electronic properties of organic linkers in coordination cages can control guest uptake and release to realize high activities and selectivities, which will promote the design of new types of molecular containers for supramolecular catalysis.

## Methods

### Synthesis of the cages

A mixture of **L**_1_ or **L**_2_ (0.24 mmol), 2-formylpyridine (102 mg, 0.96 mmol), and Zn(OTf)_2_ (116 mg, 0.32 mmol) was dissolved in 30 mL CH_2_Cl_2_ and 30 mL CH_3_CN and then was heated at 70 °C for 8 h to give a clear solution. Single crystals suitable for X-ray diffraction analysis were obtained by slow diffusion of a mixture of Et_2_O and THF or 1,4-dioxane and THF (2:1 v/v) into the solution at r.t. for 7 days. The product can be best formulated as [Zn_8_(**L**_1_)_6_]16OTf·2THF·3CH_3_CN for TPE-**1** and [Zn_8_(**L**_**2**_)_6_]16OTf·4THF·8dioxane for TPE-**2** on the basis of microanalysis, TGA, Q-TOF-MS, and IR.

TPE**-1**. Yield: 200 mg, about 64%. Anal (%). Calcd for C_336_H_252_N_52_Zn_8_S_16_O_51_F_48_: C, 59.16; H, 3.72; N, 10.68. Found: C, 59.23; H, 3.81; N, 10.86. ^1^H NMR (400 MHz, DMSO-*d*_6_) *δ*: 8.73 (bs, 24H), 8.65 (bs, 24H), 8.12 (s, 24H), 8.11 (s, 24H), 7.62 (bs, 24H), 7.20 (bs, 48H), 7.07 (bs, 48H). ^13^C NMR (100 MHz, DMSO-*d*_6_) *δ*: 160.6, 150.1, 132.3, 125.9, 122.9, 121.8, 119.7, 116.4, 113.8. IR (KBr pellet, v/cm^−1^): 1598 (m), 1513 (m), 1445 (w), 1267 (vs), 1162 (s), 1031 (s), 914 (w), 839 (w), 777 (w), 639 (m), 574 (s), 518 (m). Q-TOF-MS: m/z: 797.84 [Zn_8_(**L**_1_)_6_·8OTf·THF·2CH_3_CN]^8+^, 829.24 [Zn_8_(**L**_1_)_6_·8OTf·2THF·6CH_3_CN·H_2_O]^8+^, 963.19 [Zn_8_(**L**_1_)_6_·9OTf·4THF·CH_3_CN·2H_2_O]^7+^, 1083.96 [Zn_8_(**L**_1_)_6_·10OTf]^6+^, 1331.55 [Zn_8_(**L**_1_)_6_·11OTf]^5+^, 1700.41 [Zn_8_(**L**_1_)_6_·12OTf]^4+^.

TPE**-2**. Yield: 140 mg, about 35%. Anal (%). Calcd for C_508_H_471_N_48_Zn_8_S_16_O_62_F_48_: C, 59.95; H, 4.61; N, 6.61. Found: C, 59.89; H, 4.75; N, 6.76. ^1^H NMR (400 MHz, DMSO-*d*_6_) *δ*: 8.74 (bs, 48H), 8.15 (bs, 48H), 7.77 (bs, 24H), 7.68−7.19 (m, 192H). ^13^C NMR (100 MHz, DMSO-*d*_6_) *δ*: 160.8, 150.0, 148.9, 141.1, 139.0, 132.1, 131.8, 127.6, 127.4, 126.4, 125, 123.0, 119.6, 114.7. IR (KBr pellet, v/cm^−^^1^): 1599 (m), 1494 (s), 1445 (w), 1267 (vs), 1164 (m), 1031 (s), 1004 (w), 823 (m), 803 (m), 776 (w), 639 (m), 574 (s), 517 (w). Q-TOF-MS: m/z: 559.11 [Zn_8_(**L**_2_)_6_·3OTf]^13+^, 650.41 [Zn_8_(**L**_2_)_6_·4OTf·4(1,4-dioxane)·THF·CH_3_CN]^12+^, 741.06 [Zn_8_(**L**_2_)_6_·5OTf·5(1,4-dioxane)·THF·CH_3_CN]^11+^, 795.89 [Zn_8_(**L**_2_)_6_·6OTf·2(1,4-dioxane)·CH_3_CN]^10+^, 916.64 [Zn_8_(**L**_2_)_6_·7OTf·3(1,4-dioxane)·THF·CH_3_CN]^9+^, 1267.19 [Zn_8_(**L**_2_)_6_·9OTf·7(1,4-dioxane)·THF]^9+^.

### General procedure for the cage-based catalysis

To a solvent mixture of CH_3_CN and toluene (1:2 v/v, 3 mL), anthranilamide (0.68 mg, 0.05 mmol), aldehyde (0.055 mmol), and TPE-**1** (0.4 mg, 5 × 10^-5^ mmol) or TPE-**2** (0.5 mg, 5 × 10^-5^ mmol) were added. The resultant solution was stirred at 40 °C for 14 h. The reaction mixture was cooled down to r.t. and extracted with ethyl acetate. The ethyl acetate solution was washed with water and dried over anhydrous sodium sulfate. The solvent was removed under reduced pressure and the crude products were separated by the column on silica gel (EtOAc/petroleum ether) to get the isolated yield.

### General procedure for the multiple turnover catalysis

To a solution of the TPE**-1** (0.4 mg, 5×10^−^^5^ mmol) or TPE**-2** (0.5 mg, 5×10^−5^ mmol) in the mixed solvents of CH_3_CN (1 mL) and toluene (2 mL), we added ten times successive portions of **3a** (0.05 mmol) and **4b** (0.055 mmol), waiting until each aliquot had completely reacted before adding the next.

### General procedure for UV−Vis titration

The titration experiments were carried out by adding 30.0 μL solution of substrates (1.0×10^−3^ mol L^−^^1^) to a solution of TPE-**1** or TPE-**2** (1.0×10^−5^ mol L^−^^1^) in 3.0 mL CH_3_CN every 5 min. The absorption was measured at room temperature.

### Single-crystal X-ray crystallography

Single-crystal XRD data for TPE-**1** and TPE-**2** were collected on a Bruker D8 VENTURE CMOS photon 100 diffractometer with helios mx multilayer monochromator Cu Kα radiation (*λ* = 1.54178 Å) at 173 K. The empirical absorption correction was applied by using the SADABS program (G.M. Sheldrick, SADABS, program for empirical absorption correction of area detector data; University of Göttingen, Göttingen, Germany, 1996). The structure was solved by direct methods with SHELXS-2014 and refined with SHELXL-2014 using OLEX2-1.2. In both cages, all non-H atoms were subjected to anisotropic refinement by full matrix program. Contributions to scattering due to these highly disordered solvent molecules were removed using the SQUEEZE routine of PLATON. Structures were then refined again using the data generated. Crystal data and details of the data collection are given in Supplementary Table 1, and selected bond distances and angles are presented in Supplementary Tables 2−4.

### Molecular simulations

Molecular simulations were conducted to estimate the interaction energies for **3a** and **4** (**4b**, **4e** or **4f**) in TPE-**1** and TPE-**2**, respectively. The cages and reactants were described by the Lennard−Jones (LJ) and electrostatic potentials. The LJ potential parameters as listed in Supplementary Table 6 were adopted from the universal force field^44^, and the atomic charges of the cages and reactants were estimated using the Qeq method^45^. To calculate the interaction energy for **3a** + **4** in each cage, first, **3a** and **4** together were inserted into TPE-**1** or TPE-**2**, and followed by optimization using Materials Studio (Accelrys Inc., 2008); then, MD simulation was performed at 313 K for 15 ns. The cage structure was assumed to be rigid during MD simulation, but all the reactant molecules were flexible and free to move. The LJ interactions were evaluated with a cutoff of 12 Å, and the electrostatic interactions were estimated using the Ewald summation method with an accuracy of 10^−3^ kcal mol^−^^1^.

## Electronic supplementary material

Supplementary Information

## Data Availability

The X-ray crystallographic coordinates for the structures reported in this article have been deposited at the Cambridge Crystallographic Data Centre (CCDC), under deposition numbers CCDC 1858566, 1858567, and 1858587. These data can be obtained free of charge from The Cambridge Crystallographic Data Centre via www.ccdc.cam.ac.uk/data_request/cif. All other data supporting the findings of this study are available within the article and its Supplementary Information, or from the corresponding author upon reasonable request.
