# Supplementary material for: Design and self-assembly of hexahedral coordination cages for cascade reactions
Source: Nat Commun. 2018 Oct 24;9:4423. doi: 10.1038/s41467-018-06872-0 (PMC6200784; doi:10.1038/s41467-018-06872-0)
Supplement: Supplementary file 1 — Supplementary Information [file 41467_2018_6872_MOESM1_ESM.pdf]

Supporting Information for

Design and self-assembly of hexahedral coordination cages for cascade reactions

Jiao et al.

Supplementary Methods

Synthesis of ligands

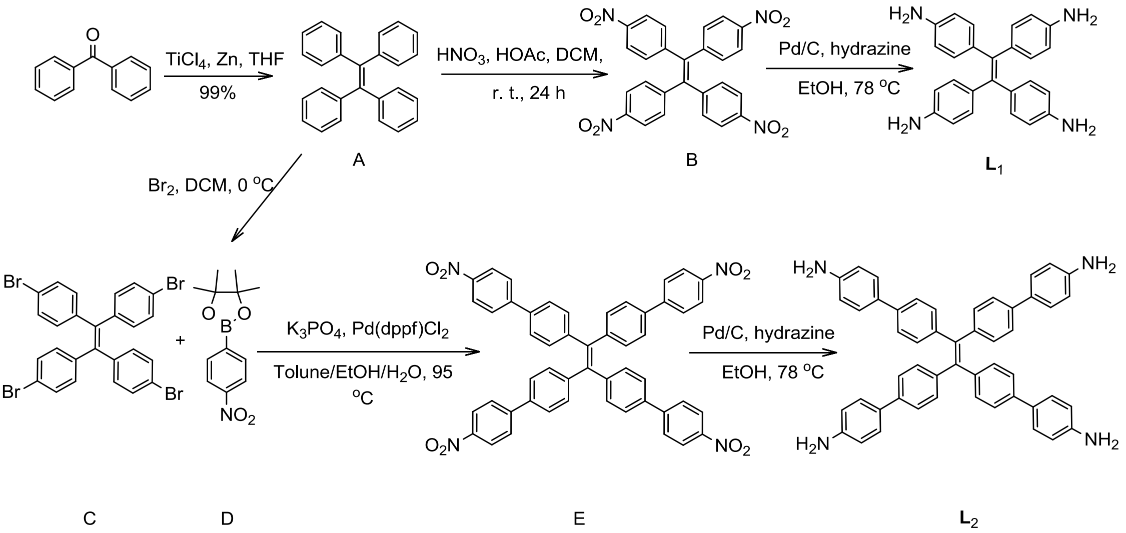

Synthesis of tetraphenylethene (A)

Tetraphenylethene was synthesized according to the standard procedure.[3] A suspension of benzophenone (5 g, 30 mmol) and zinc (5.9 g, 90 mmol) in 300 mL dried THF was stirred under nitrogen at 0 °C. $TiCl_4$ (5 mL, 60 mmol) was injected slowly over a period of 30 min. The ice-water-bath was removed and the mixture was stirred at 70 °C for 12 h. The reaction was quenched by aqueous solution of potassium carbonate and extracted with ethyl acetate (2 × 200 mL). The organic phase was combined, washed with brine, and dried over anhydrous sodium sulfate. After filtration, the filtrate was concentrated under vacuum to afford **A** as a white solid (5 g, 99%) ^{1}H NMR (400 MHz, $CDCl_3$) δ: 7.02 (m, 8H), 7.09-7.11 (m, 12H).

Synthesis of 1,1,2,2-tetrakis(4-nitrophenyl)ethene (B)[4]

A (5 g, 15 mmol) was dissolved in a mixed solvent of acetic acid and dichloromethane (30 mL/60 mL). 30 mL concentrated nitric acid was added slowly to the reaction mixture, and the mixture was stirred for 16 h at room temperature. The reaction mixture was extracted with dichloromethane (2 × 200 mL), and the combined organic phase was washed with water, and dried over anhydrous Na_2SO_4. After filtration, the filtrate was concentrated to afford crude product, which was recrystallized from 1,4-dioxane to give **B** as a light yellow solid (4.6 g, 60%). ^{1}H NMR (400 MHz, DMSO-d_6) δ: 8.12 (d, J = 8.7 Hz, 8H), 7.35 (d, J = 8.7, 8H).

Synthesis of 4,4',4'',4'''-(ethene-1,1,2,2-tetrayl)tetraaniline (L_1)

A 250 mL flame-dried round bottom flask was charged with **B** (2.5 g, 5 mmol), Pd/C (10%, 450 mg), hydrazine hydrate (23 mL, 300 mmol), and ethanol (120 mL). The mixture was degassed for three times, and then the reaction mixture was stirred at 78 °C for 12 h. Then the mixture was filtered and the filtrate was concentrated. After addition of dichloromethane (200 mL), the organic phase was washed with water and dried over anhydrous Na_2SO_4. After filtration, the filtrate was concentrated to give

crude product, which was purified by column chromatography on silica gel (DCM/EtOAc, 5:1 v/v) to afford **L**$_1$ as a yellow solid (1.2 g, 60%). ^{1}H NMR (400 MHz, DMSO-d_6) δ: 6.60(d, J = 8.5 Hz, 8H), 6.27 (d, J = 8.5 Hz, 8H), 4.97 (s, 8H)

Synthesis of 1,1,2,2-tetrakis(4-bromophenyl)ethene (C)[4]

A (5 g, 15 mmol) was dissolved in a mixed solvent of dichloromethane and acetic acid (30 mL: 20 mL) at 0 ^{o}C. With vigorous stirring, bromine (7.5 mL, 150 mmol) was added slowly. After completion of the addition, the mixture was stirred at 50 ^{o}C for 4 h. Pour the reaction mixture into ice-water, and the precipitate was collected by filtration and washed with water and ethanol. The white solid was dried in 65 ^{o}C oven for 8 h to afford pure **C** (8.3 g, 85%). ^{1}H NMR (400 MHz, $CDCl_3$) δ: 7.27 (d, J = 8.4 Hz, 8H) and 6.85 (d, J = 8.4 Hz, 8H).

Synthesis of 1,1,2,2-tetrakis(4'-nitro-[1,1'-biphenyl]-4-yl)ethene (E)

A mixture of **C** (2 g, 3 mmol), (4-nitrophenyl)boronic acid pinacol ester (**D**, 3.8 g, 15.3 mmol), K_3PO_4 (8.3 g, 39 mmol) and Pd(dppf)Cl_2 (260 mg, 0.28 mmol) in toluene/EtOH/H_2O (3:1:1 v/v/v, 100 mL) was degassed for three times and the suspension was stirred under N_2 at 95 ^{o}C for 10 h. After cooling to r.t., the mixture was concentrated and DCM (200mL) was added. The combined organic phase was washed with brine, dried over Na_2SO_4 and then concentrated under reduced pressure. The crude product was purified by column chromatography on silica gel (hexane/EtOAc, 10:1 v/v) to afford **E** as a yellow solid (yield 40%). ^{1}H NMR (400 MHz, $CDCl_3$) δ: 8.25 (d, J = 8.6 Hz,8H), 7.70 (d, J = 8.6 Hz, 8H), 7.47 (d, J = 8.1 Hz, 8H), 7.26 (d, J = 6.7 Hz, 8H) ^{13}C NMR (400 MHz, $CDCl_3$) δ: 147.3, 146.9, 144.1, 137.3, 132.4, 127.7, 127.2, 124.3.

Synthesis of 4',4''',4''''',4'''''''-(ethene-1,1,2,2-tetrayl)tetrakis(([1,1'-biphenyl]-4-amine)) (L$_2$)

A 250 mL round bottom flask was charged with **E** (2 g, 2.5 mmol), Pd/C (10%, 225 mg), hydrazine hydrate (11.5 mL, 150 mmol), and ethanol (60 mL). The mixture was degassed for three times, and then the reaction mixture was stirred at 78 ^{o}C for 12 h. Then the mixture was filtered and the filtrate was concentrated. After addition of dichloromethane (200 mL), the organic phase was washed with water and dried over anhydrous Na_2SO_4. After filtration, the filtrate was concentrated to give crude product, which was purified by column chromatography on silica gel (DCM/EtOAc, 5:1 v/v) to afford **L**$_2$ as a yellow solid (1.0 g, 60%). H NMR (400 MHz, DMSO-d_6) δ: 7.35 (dd, J = 13.1, 8.3 Hz, 16H), 7.04 (d, J = 8.2 Hz, 8H), 6.58 (d, J = 8.3 Hz, 8H), 5.20 (s, 8H) ^{13}C NMR (100 MHz, DMSO-d_6) δ: 148.8, 141.6, 139.9, 138.7, 131.9, 127.3, 127.0, 124.7, 114.6. IR (KBr pellet, v/cm^{-1}): 3381 (m), 1619 (s), 1527 (m), 1497 (s), 1400 (w), 1284 (m), 1182(w), 1046 (w), 823 (m), 804 (w), 683 (s), 540 (s), 502 (s). ESI-MS: m/z 349.34 (Calcd m/z 349.17 for [**L**$_2$ + 2H]$^{2+}$)

Synthesis of Zn(PI)$_3$

A mixture of *p*-Toluidine (53 mg, 0.49 mmol), prydine-2-formyaldehyde (53 mg, 0.49

mmol), $Zn(OTf)_2$ (44 mg, 0.12 mmol), 20 mL acetonitrile was heated at 40 ^oC for 12 h. Crystals of $Zn(PI)_3$ were obtained by diffusion of elthyl ether into the reaction mixture at room temperature for 3 days. ^1H NMR (400 MHz, DMSO-d_6) δ: 8.76 (d, J = 33.3 Hz, 2H), 8.11 (d, J = 7.0 Hz, 2H), 7.73 (s, 1H), 7.19 (s, 4H), 2.30 (s, 3H). ^{13}C NMR (100 MHz, DMSO-d_6) δ: 160.54, 150.05, 130.18, 129.69, 123.03 - 122.81, 122.32, 119.56, 21.07. IR (KBr pellet, v/cm^{-1}): 1629 (s), 1595 (vs), 1507 (s), 1482 (m), 1446 (m), 1267 (vs), 1153 (s), 1029 (s), 913 (m), 818 (s), 776 (m), 638 (s), 517 (m).

Supplementary Table 1. Crystal data and structure refinement for TPE-1, TPE-2 and Zn(PI)$_3$.

Identification code	TPE-**1**	TPE-**2**	Zn(PI)$_3$
Empirical formula	$C_{300}H_{216}N_{48}Zn_8$	$C_{457}H_{312}N_{48}F_{39}O_{39}S_{13}$	$C_{41}H_{36}F_6N_6O_6S_2Zn$
Formula weight	5016.16	8780.27	952.25
Temperature (K)	173(2) K	173(2) K	293(2) K
Wavelength (Å)	1.54178	1.54178	0.71073
Crystal system, space group	Hexagonal, $P6_222$	Triclinic, $P\bar{1}$	Monoclinic, $P2_1/c$
Unit cell dimensions	a = 24.2986(11) Å;<br>b = 24.2986(11) Å;<br>c = 64.789(6) Å;<br>α = 90 °;<br>β = 90 °;<br>γ = 120 °	a = 26.9878(12) Å;<br>b = 33.3614(16) Å;<br>c = 45.907(2) Å;<br>α = 83.588(2) °;<br>β = 79.043(3) °;<br>γ = 81.813(2) °	a = 21.654(6) Å;<br>b = 9.814(2) Å;<br>c = 22.472(6) Å;<br>α = 90 °;<br>β = 116.549(4) °;<br>γ = 90 °
Volume (Å^3), Z	33139(4), 3	40013(3), 2	4271(2), 4
Density (calculated)	0.754	0.729	1.481
Absorption	0.776	0.979	0.753
F(000)	7776	9002	1952
θ range for data	3.443 to 50.494 deg	2.054 to 36.520 deg	1.051 to 25.116 deg
Limiting indices	$-24 \leq h \leq 24$<br>$-23 \leq k \leq 24$<br>$-64 \leq l \leq 64$	$-20 \leq h \leq 20$<br>$-25 \leq k \leq 25$<br>$-35 \leq l \leq 35$	$-25 \leq h \leq 22$<br>$-11 \leq k \leq 11$<br>$-26 \leq l \leq 26$
Reflections collected	176714	109954	28242
Independent reflections	11548[R(int)=0.0700]	36092[R(int)=0.0495]	7511 [R(int) =0.0612]
Completeness	99.3 %	93.6 %	98.5%
Refinement method	Full-matrix least-squares	Full-matrix least-squares	Full-matrix least-squares
Data/restraints/ parameters	11548 / 1408 / 659	36092 / 9602 / 4573	7511 / 0 / 562
Goodness-of-fit on F^2	1.044	1.282	1.049
Final R indices [I>2sigma(I)]	R_1 = 0.0614,<br>wR_2 = 0.1848	R_1 = 0.1569,<br>wR_2 = 0.4550	R_1 = 0.0707,<br>wR_2 = 0.2066
R indices (all data)	R_1 = 0.0864,<br>wR_2 = 0.2149	R_1 = 0.1878,<br>wR_2 = 0.5004	R_1 = 0.1209,<br>wR_2 = 0.2768
Largest diff. peak and hole	0.235 and -0.389 e.A^{-3}	1.283 and -0.821 e.A^{-3}	0.775 and -0.762 e.A^{-3}

Supplementary Table 2. Selected bond lengths [Å] and angles [°] for TPE-1.

Zn(1)-N(1)	2.105(6)	Zn(2)-N(7)	2.188(9)
Zn(1)-N(2)	2.152(8)	Zn(2)-N(8)	2.096(6)
Zn(1)-N(3)	2.278(8)	Zn(2)-N(9)	2.239(8)
Zn(1)-N(4)	2.119(6)	Zn(2)-N(10)	2.145(6)
Zn(1)-N(5)	2.162(8)	Zn(2)-N(11)#1	2.179(5)
Zn(1)-N(6)	2.163(6)	Zn(2)-N(12)#1	2.106(8)
N(5)-Zn(1)-N(3)	96.6(3)	N(12)#1-Zn(2)-N(10)	88.5(3)
N(5)-Zn(1)-N(6)	75.7(3)	N(12)#1-Zn(2)-N(7)	93.5(3)
N(4)-Zn(1)-N(2)	169.0(3)	N(12)#1-Zn(2)-N(11)	76.5(3)
N(4)-Zn(1)-N(6)	96.2(3)	N(10)-Zn(2)-N(9)	74.8(3)
N(2)-Zn(1)-N(3)	94.2(3)	N(10)-Zn(2)-N(7)	170.1(3)
N(2)-Zn(1)-N(5)	94.5(3)	N(10)-Zn(2)-N(11)#1	95.9(3)
N(2)-Zn(1)-N(6)	94.8(3)	N(7)-Zn(2)-N(9)	95.4(3)
N(6)-Zn(1)-N(3)	168.6(3)	N(8)-Zn(2)-N(9)	94.4(3)
N(1)-Zn(1)-N(3)	92.2(3)	N(8)-Zn(2)-N(12)#1	164.1(3)
N(1)-Zn(1)-N(5)	169.5(3)	N(8)-Zn(2)-N(10)	103.2(3)
N(1)-Zn(1)-N(4)	100.4(3)	N(8)-Zn(2)-N(7)	76.8(3)
N(1)-Zn(1)-N(2)	79.1(3)	N(8)-Zn(2)-N(11)#1	91.4(3)
N(1)-Zn(1)-N(6)	96.4(3)	N(11)#1-Zn(2)-N(9)	169.9(3)
N(12)#1-Zn(2)-N(	99.1(3)	N(11)#1-Zn(2)-N(7)	93.9(3)

Symmetry transformations used to generate equivalent atoms:

#1 x-y,-y,-z #2 -x,-y,z #3 -x+y,y,-z

Supplementary Table 3. Selected bond lengths [Å] and angles [°] for TPE-2.

Zn(1)-N(3)	2.180(14)	Zn(5)-N(16)	2.168(16)
Zn(1)-N(4)	2.266(16)	Zn(5)-N(19)	2.204(10)
Zn(1)-N(11)	2.127(9)	Zn(5)-N(24)	2.233(17)
Zn(1)-N(12)	2.132(10)	Zn(5)-N(27)	2.138(11)
Zn(1)-N(18)	2.167(10)	Zn(5)-N(40)	2.190(17)
Zn(1)-N(36)	2.223(15)	Zn(5)-N(44)	2.199(12)
Zn(2)-N(1)	2.171(9)	Zn(6)-N(17)	2.149(10)
Zn(2)-N(6)	2.161(8)	Zn(6)-N(21)	2.156(12)
Zn(2)-N(7)	2.181(14)	Zn(6)-N(22)	2.187(17)
Zn(2)-N(8)	2.224(14)	Zn(6)-N(26)	2.179(12)
Zn(2)-N(10)	2.263(14)	Zn(6)-N(30)	2.245(18)
Zn(2)-N(31)	2.165(9)	Zn(6)-N(37)	2.141(17)
Zn(3)-N(2)	2.166(15)	Zn(7)-N(29)	2.180(19)
Zn(3)-N(13)	2.109(10)	Zn(7)-N(32)	2.231(13)
Zn(3)-N(15)	2.199(16)	Zn(7)-N(34)	2.23(2)
Zn(3)-N(23)	2.189(11)	Zn(7)-N(41)	2.11(2)
Zn(3)-N(33)	2.214(10)	Zn(7)-N(43)	2.089(13)
Zn(3)-N(39)	2.272(15)	Zn(7)-N(46)	2.144(13)
Zn(4)-N(5)	2.255(14)	Zn(8)-N(28)	2.30(2)
Zn(4)-N(9)	2.218(15)	Zn(8)-N(35)	2.239(18)
Zn(4)-N(14)	2.196(9)	Zn(8)-N(42)	2.147(12)
Zn(4)-N(20)	2.137(10)	Zn(8)-N(45)	2.117(16)
Zn(4)-N(25)	2.148(9)	Zn(8)-N(47)	2.24(3)
Zn(4)-N(38)	2.193(15)	Zn(8)-N(48)	2.153(16)
N(3)-Zn(1)-N(4)	96.3(6)	N(16)-Zn(5)-N(19)	173.3(6)
N(3)-Zn(1)-N(36)	100.0(5)	N(16)-Zn(5)-N(24)	103.2(6)
N(11)-Zn(1)-N(3)	76.1(5)	N(16)-Zn(5)-N(40)	103.7(6)
N(11)-Zn(1)-N(4)	89.4(5)	N(16)-Zn(5)-N(44)	91.1(6)
N(11)-Zn(1)-N(12)	101.0(5)	N(19)-Zn(5)-N(24)	83.5(5)
N(11)-Zn(1)-N(18)	95.3(5)	N(27)-Zn(5)-N(16)	78.4(6)
N(11)-Zn(1)-N(36)	175.0(5)	N(27)-Zn(5)-N(19)	95.0(5)
N(12)-Zn(1)-N(3)	97.8(5)	N(27)-Zn(5)-N(24)	169.5(6)
N(12)-Zn(1)-N(4)	164.1(5)	N(27)-Zn(5)-N(40)	97.7(6)
N(12)-Zn(1)-N(18)	93.1(5)	N(27)-Zn(5)-N(44)	92.5(6)
N(12)-Zn(1)-N(36)	76.3(6)	N(40)-Zn(5)-N(19)	76.1(6)
N(18)-Zn(1)-N(3)	167.2(5)	N(40)-Zn(5)-N(24)	92.0(6)
N(18)-Zn(1)-N(4)	73.8(6)	N(40)-Zn(5)-N(44)	163.4(6)
N(18)-Zn(1)-N(36)	89.1(5)	N(44)-Zn(5)-N(19)	90.1(5)
N(36)-Zn(1)-N(4)	94.2(6)	N(44)-Zn(5)-N(24)	77.1(7)
N(1)-Zn(2)-N(7)	89.2(4)	N(17)-Zn(6)-N(21)	96.8(5)
N(1)-Zn(2)-N(8)	77.4(5)	N(17)-Zn(6)-N(22)	92.3(5)

N(1)-Zn(2)-N(10)	171.5(5)	N(17)-Zn(6)-N(26)	95.6(5)
N(6)-Zn(2)-N(1)	92.2(4)	N(17)-Zn(6)-N(30)	167.8(7)
N(6)-Zn(2)-N(7)	77.6(5)	N(21)-Zn(6)-N(22)	77.9(7)
N(6)-Zn(2)-N(8)	167.0(5)	N(21)-Zn(6)-N(26)	96.8(6)
N(6)-Zn(2)-N(10)	91.8(4)	N(21)-Zn(6)-N(30)	91.5(6)
N(6)-Zn(2)-N(31)	94.4(4)	N(22)-Zn(6)-N(30)	98.2(7)
N(7)-Zn(2)-N(8)	94.4(5)	N(26)-Zn(6)-N(22)	171.0(6)
N(7)-Zn(2)-N(10)	99.0(5)	N(26)-Zn(6)-N(30)	74.4(7)
N(8)-Zn(2)-N(10)	99.6(5)	N(37)-Zn(6)-N(17)	76.0(6)
N(31)-Zn(2)-N(1)	95.2(5)	N(37)-Zn(6)-N(21)	169.4(6)
N(31)-Zn(2)-N(7)	171.1(5)	N(37)-Zn(6)-N(22)	94.4(6)
N(31)-Zn(2)-N(8)	94.2(5)	N(37)-Zn(6)-N(26)	91.7(6)
N(31)-Zn(2)-N(10)	77.1(5)	N(37)-Zn(6)-N(30)	96.9(7)
N(2)-Zn(3)-N(15)	102.6(6)	N(29)-Zn(7)-N(32)	92.1(6)
N(2)-Zn(3)-N(23)	170.8(6)	N(29)-Zn(7)-N(34)	96.6(8)
N(2)-Zn(3)-N(33)	92.3(5)	N(32)-Zn(7)-N(34)	170.3(7)
N(2)-Zn(3)-N(39)	99.6(5)	N(41)-Zn(7)-N(29)	97.9(8)
N(13)-Zn(3)-N(2)	77.1(6)	N(41)-Zn(7)-N(32)	76.3(7)
N(13)-Zn(3)-N(15)	170.5(5)	N(41)-Zn(7)-N(34)	98.2(7)
N(13)-Zn(3)-N(23)	95.2(6)	N(41)-Zn(7)-N(46)	169.1(8)
N(13)-Zn(3)-N(33)	94.4(5)	N(43)-Zn(7)-N(29)	171.6(8)
N(13)-Zn(3)-N(39)	95.2(5)	N(43)-Zn(7)-N(32)	94.4(7)
N(15)-Zn(3)-N(33)	76.2(5)	N(43)-Zn(7)-N(34)	77.3(8)
N(15)-Zn(3)-N(39)	94.1(6)	N(43)-Zn(7)-N(41)	88.7(7)
N(23)-Zn(3)-N(15)	85.9(6)	N(43)-Zn(7)-N(46)	96.5(7)
N(23)-Zn(3)-N(33)	93.2(5)	N(46)-Zn(7)-N(29)	77.8(8)
N(23)-Zn(3)-N(39)	76.0(6)	N(46)-Zn(7)-N(32)	93.8(7)
N(33)-Zn(3)-N(39)	166.2(5)	N(46)-Zn(7)-N(34)	92.3(7)
N(9)-Zn(4)-N(5)	100.9(5)	N(35)-Zn(8)-N(28)	95.0(8)
N(14)-Zn(4)-N(5)	165.4(5)	N(42)-Zn(8)-N(28)	75.5(7)
N(14)-Zn(4)-N(9)	91.9(5)	N(42)-Zn(8)-N(35)	165.6(8)

Supplementary Table 4. Selected bond lengths [Å] and angles [°] for $Zn(PI)_3$.

Zn(1)-N(4)	2.216(4)	Zn(1)-N(3)	2.159(5)
Zn(1)-N(1)	2.154(5)	Zn(1)-N(6)	2.166(5)
Zn(1)-N(5)	2.237(5)	Zn(1)-N(2)	2.176(5)
N(4)-Zn(1)-N(5)	81.75(16)	N(3)-Zn(1)-N(6)	164.36(18)
N(1)-Zn(1)-N(4)	104.49(16)	N(3)-Zn(1)-N(2)	102.43(19)
N(1)-Zn(1)-N(5)	168.59(17)	N(6)-Zn(1)-N(4)	91.91(17)
N(1)-Zn(1)-N(3)	97.84(18)	N(6)-Zn(1)-N(5)	75.76(19)
N(1)-Zn(1)-N(6)	94.30(18)	N(6)-Zn(1)-N(2)	89.75(19)
N(1)-Zn(1)-N(2)	77.61(17)	N(2)-Zn(1)-N(4)	177.21(17)
N(3)-Zn(1)-N(4)	75.55(17)	N(2)-Zn(1)-N(5)	96.50(18)
N(3)-Zn(1)-N(5)	92.95(18)		

Supplementary Table 5. A comparison of reported acceleration rates for the cage promoted catalysis (Only some typical reports are listed).

Cage	Catalysis reaction	k_{cat}/k_{uncat}
$Ga_4\mathbf{L}_6$[5]	Nazarov cyclization	2.1×10^6
$Co_8\mathbf{L}_{12}$[6]	Kemp elimination	2.0×10^5
$Ga_4\mathbf{L}_6$[7]	Reductive imination	1.9×10^7
$Ga_4\mathbf{L}_6$[8]	Aza Cope rearrangement	184
$Ga_4\mathbf{L}_6$[9]	Aza-Cope electrocyclization	854
$Ga_4\mathbf{L}_6$[10]	Ester hydrolysis	3900
$Ga_4\mathbf{L}_6$[11]	Orthoformate hydrolysis	890
$Fe_4\mathbf{L}_6$[12]	Organophosphates hydrolysis	1
$Zn_8\mathbf{L}_6$	Sequential condensation and cyclization	3.8×10^4

Supplementary Table 6. Lennard-Jones parameters.

Atom	ε/k_B [K]	σ [Å]
Zn	62.39	2.46
C	52.83	3.43
H	22.14	2.57
O	30.19	3.12
N	34.72	3.26
F	25.16	3.00

Supplementary Table 7. Binding energies of different reactants in TPE-1 and TPE-2.

Reactants	Binding energy (kcal/mol)	
	@TPE-**1**	@TPE-**2**
**3a** + **4b**	-34.97	-126.95
**3a** + **4e**	44.39	-94.65
**3a** + **4f**	52.67	18.64

a

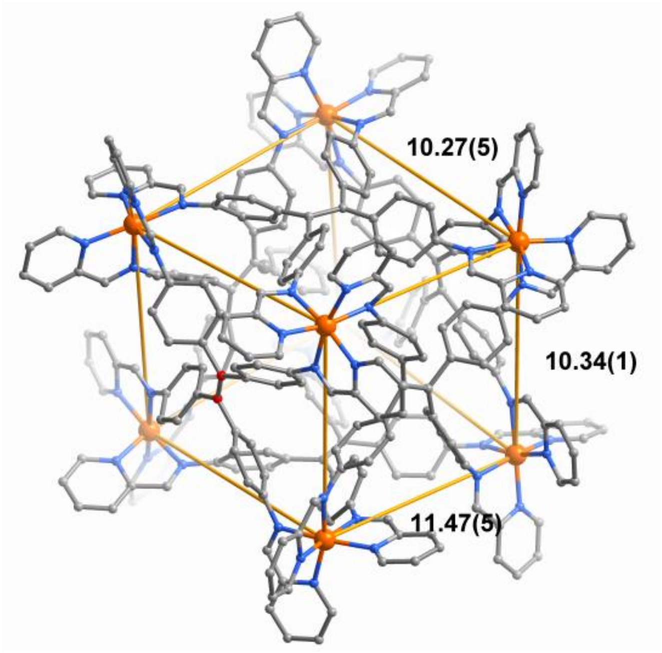

b

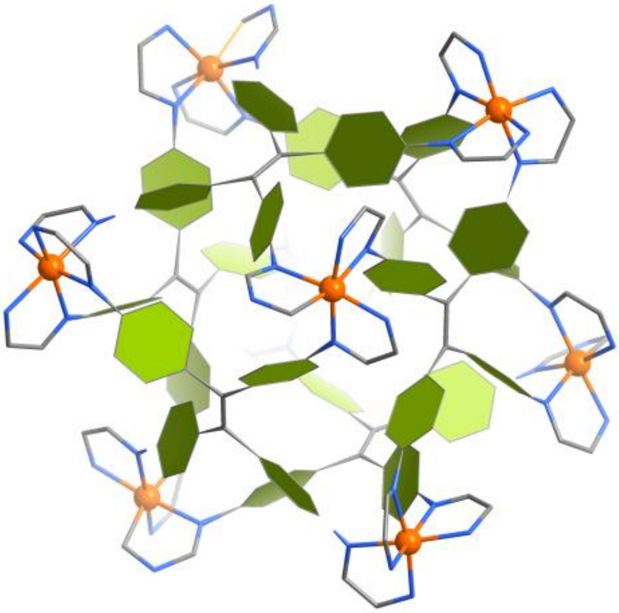

Supplementary Figure 1. (a) The skeleton of the cubic cage showing An-Zn distances along the cubic edges in TPE-1 and (b) the configuration of TPE ligand in TPE-1 (orange Zn, blue N, gray C, green hexagon benzene ring).

a

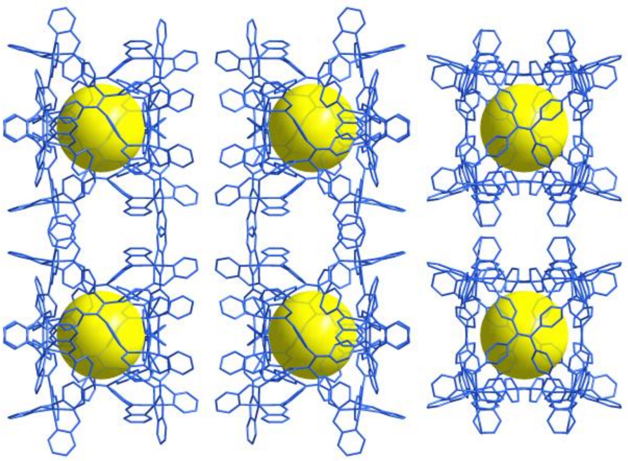

b

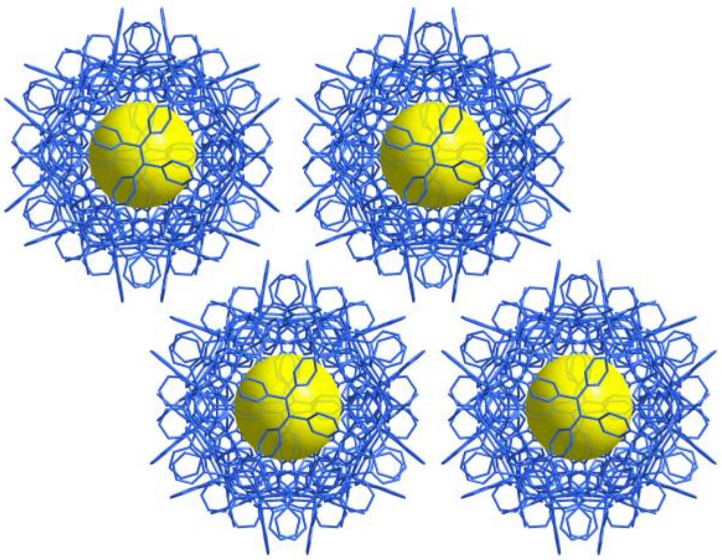

Supplementary Figure 2. View of the 3D packing mode of TPE-1 (a) along the a-axis and (b) the c-axis.

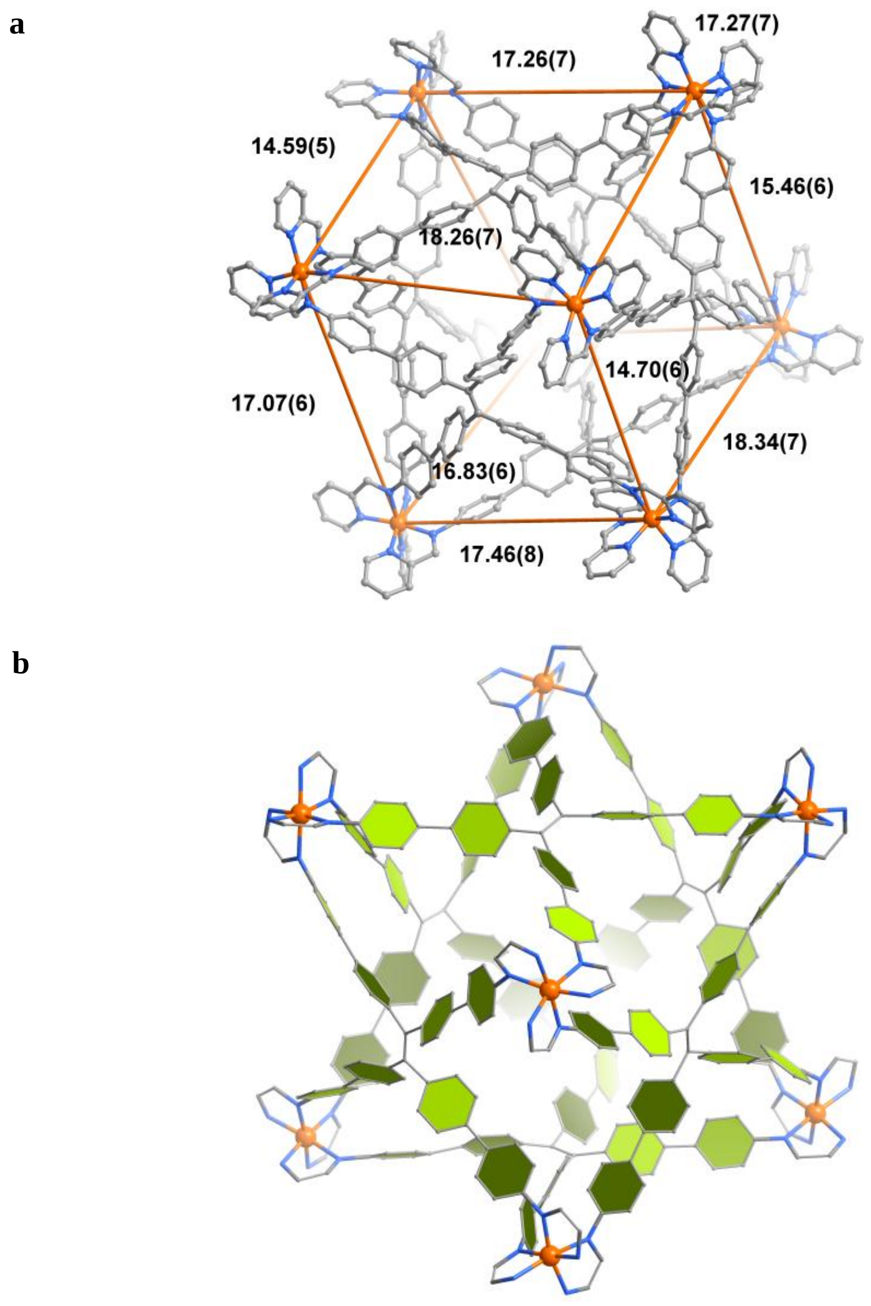

Supplementary Figure 3. (a) The skeleton of the cage showing Zn-Zn distances along the cubic edges in TPE-2 and (b) The configuration of TPE ligand in TPE-2 (orange Zn, blue N, gray C, green hexagon benzene ring).

a

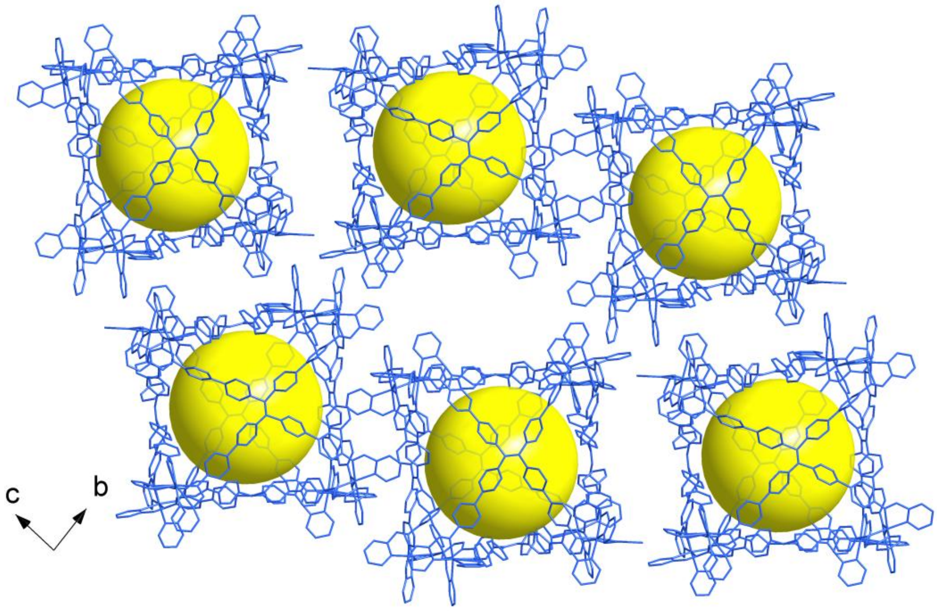

b

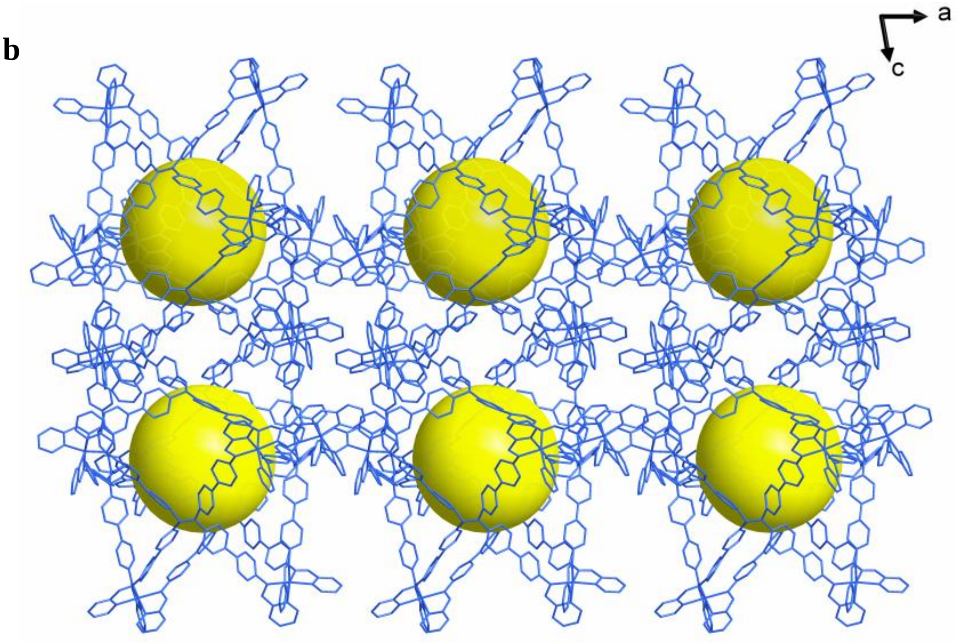

Supplementary Figure 4. View of the 3D packing mode of TPE-2 (a) along the a-axis and (b) along the b-axis.

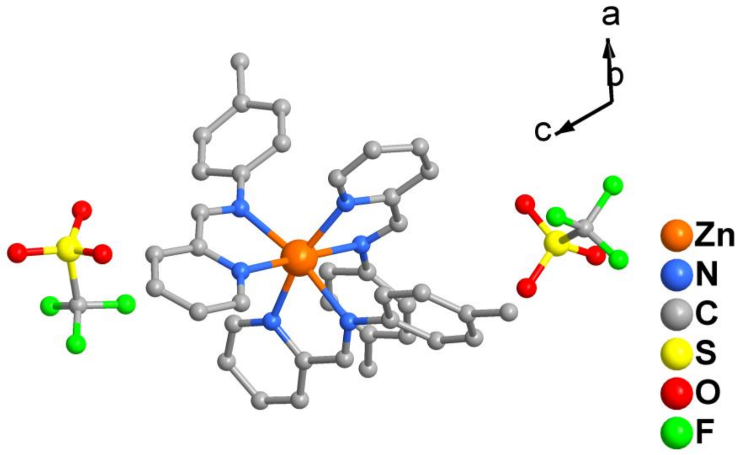

Supplementary Figure 5. The crystallographic structure of Zn(PI)$_3$.

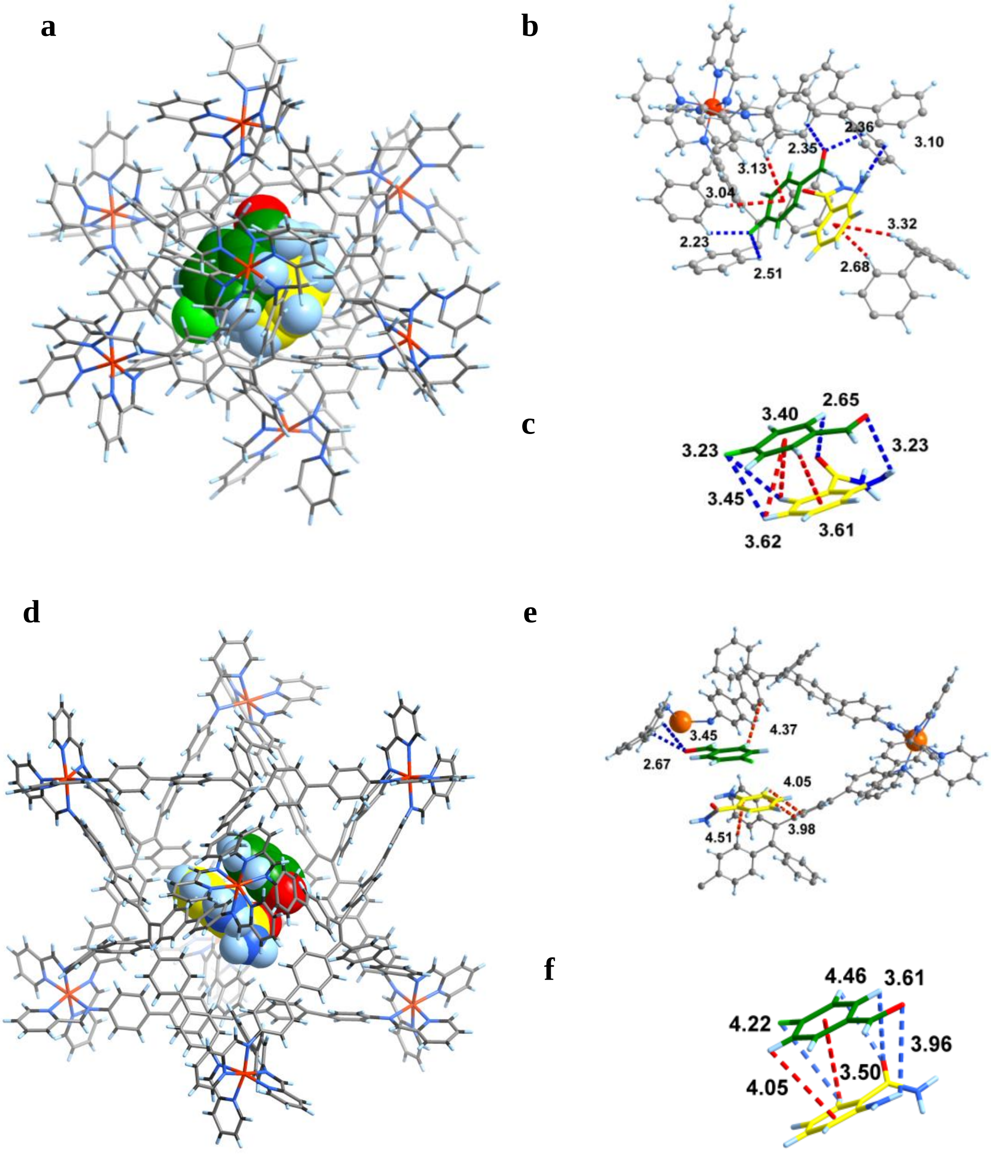

Supplementary Figure 6. A view of face-to-face stacking of the substrates 3a and 4b encapsulated in (a) TPE-1, (d) TPE-2 from molecular modeling. Views of CH-π interactions (red) and other weak interactions (blue) between TPE-1 (b, c)/ TPE-2 (e, f) and the substrates and between 3a (yellow) and 4b (green) (separations are given in Ǻ).

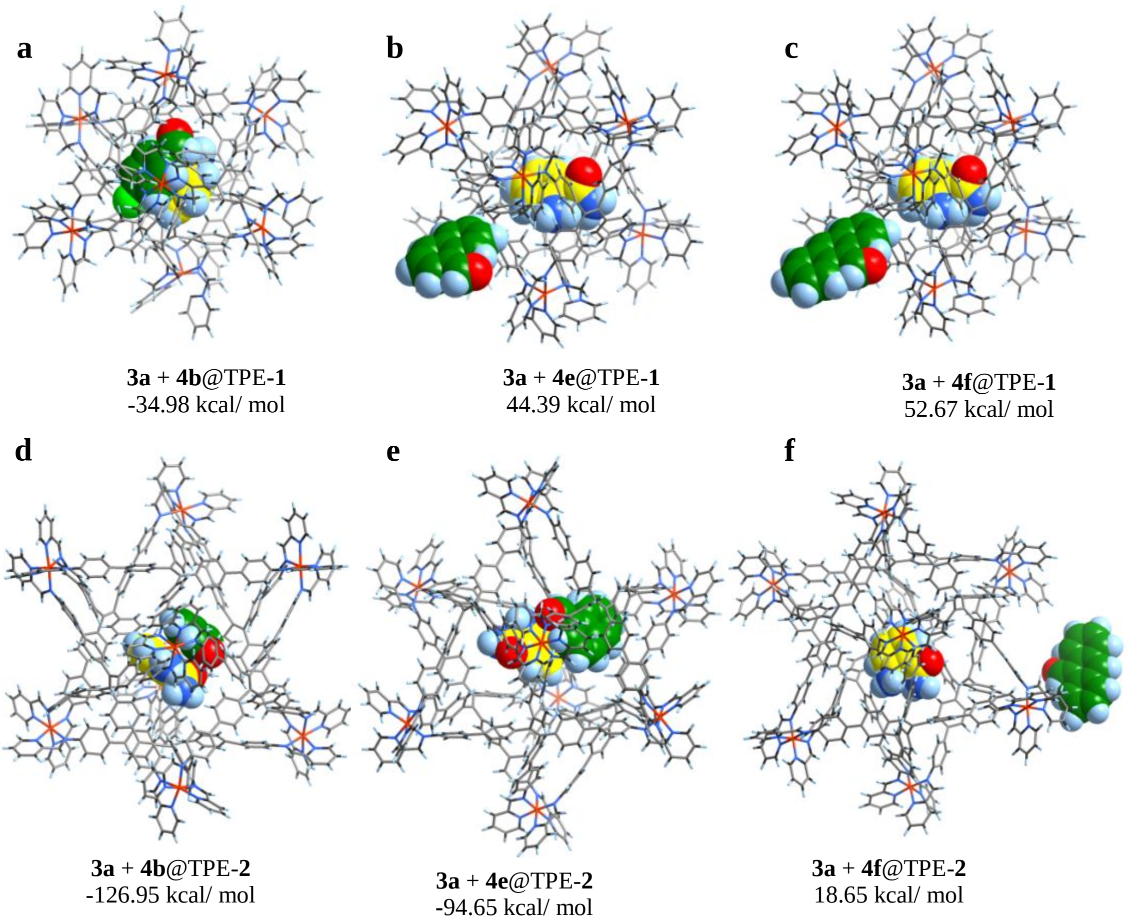

Supplementary Figure 7. Calculated interaction energies for the reactants 3a and 4 in the cavities of the cages: (a) 3a + 4b@TPE-1, (b) 3a + 4e@TPE-1, (c) 3a + 4f@TPE-1, (d) 3a + 4b@TPE-2, (e) 3a + 4e@TPE-2 and (f) 3a + 4f@TPE-2.

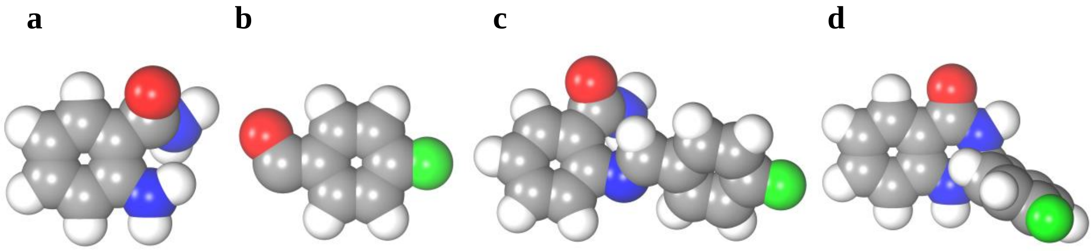

Supplementary Figure 8. Optimized geometries of reactants or products by Material Studio. (a) 3a, (b) 4b, (c) 5b and (d) 6b (Color code: N blue, C gray, O red, F cyan, H white).

a b c d

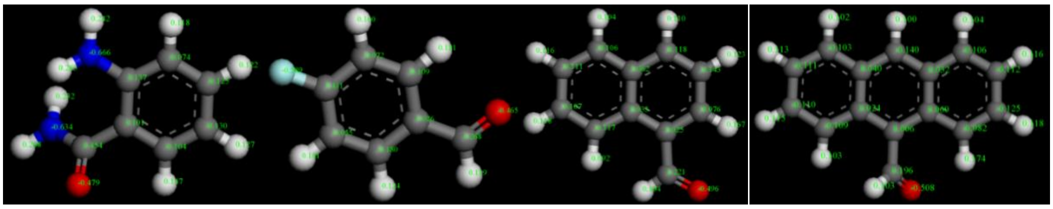

Supplementary Figure 9. Atomic charges of reactants (a) 3a, (b) 4b, (c) 4e and (d) 4f. (Color code: N blue, C gray, O red, F cyan, H white).

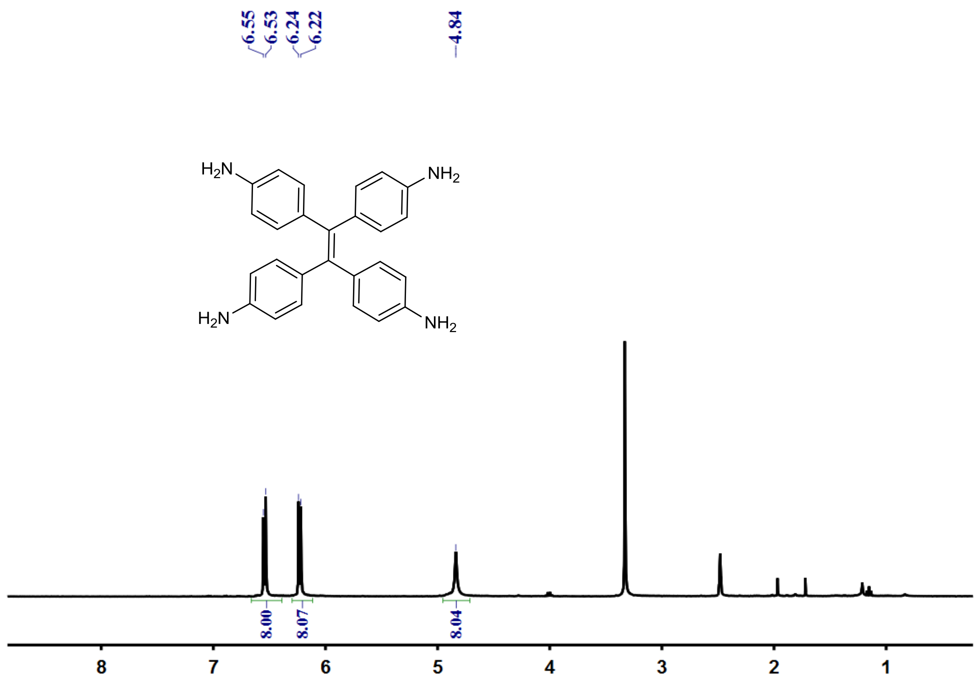

Supplementary Figure 10. ^{1}H NMR spectra of L_1 in DMSO-d_6.

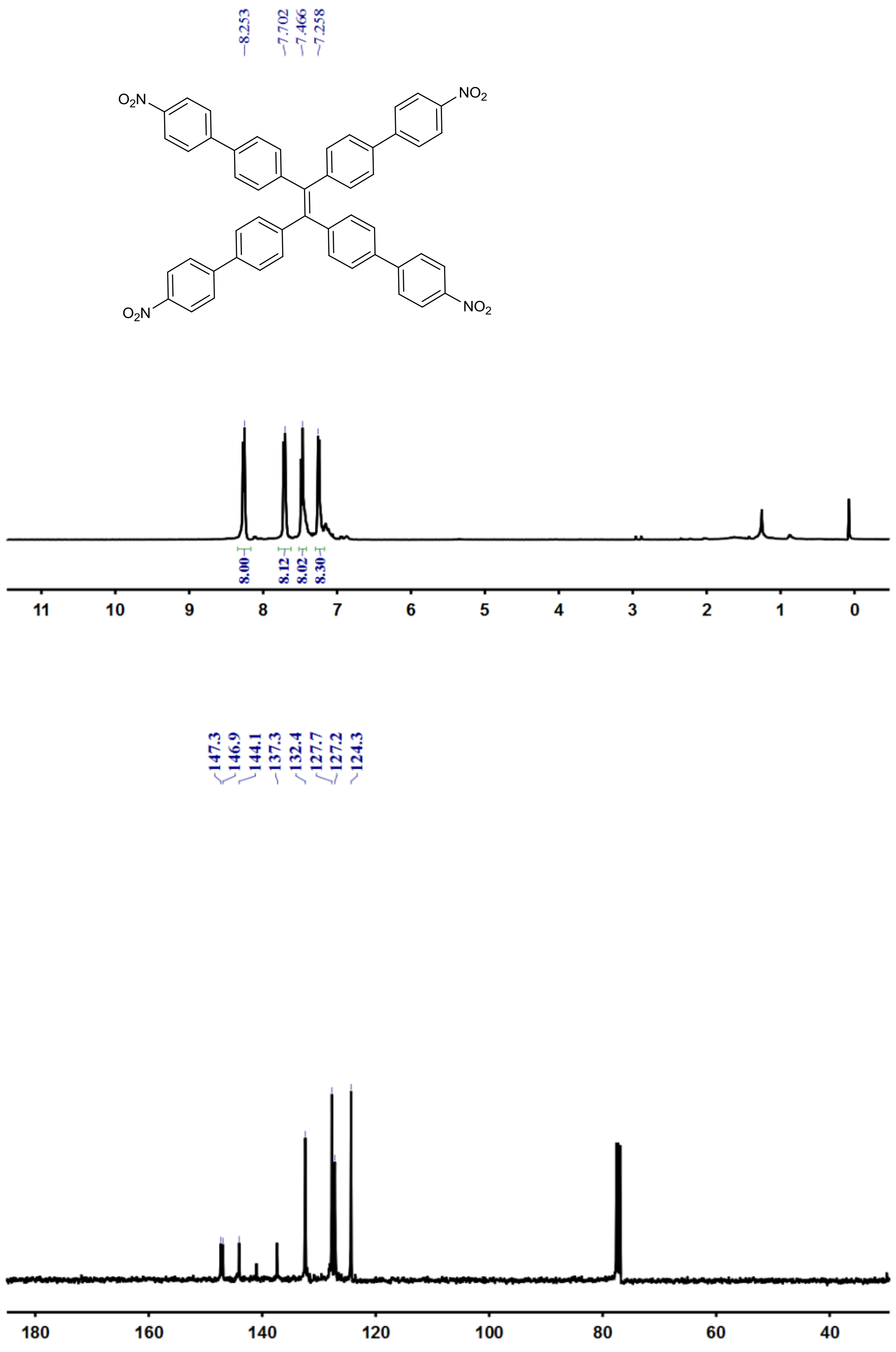

Supplementary Figure 11. **^{1}H NMR and ^{13}C NMR spectra of compound E in $CDCl_3$.**

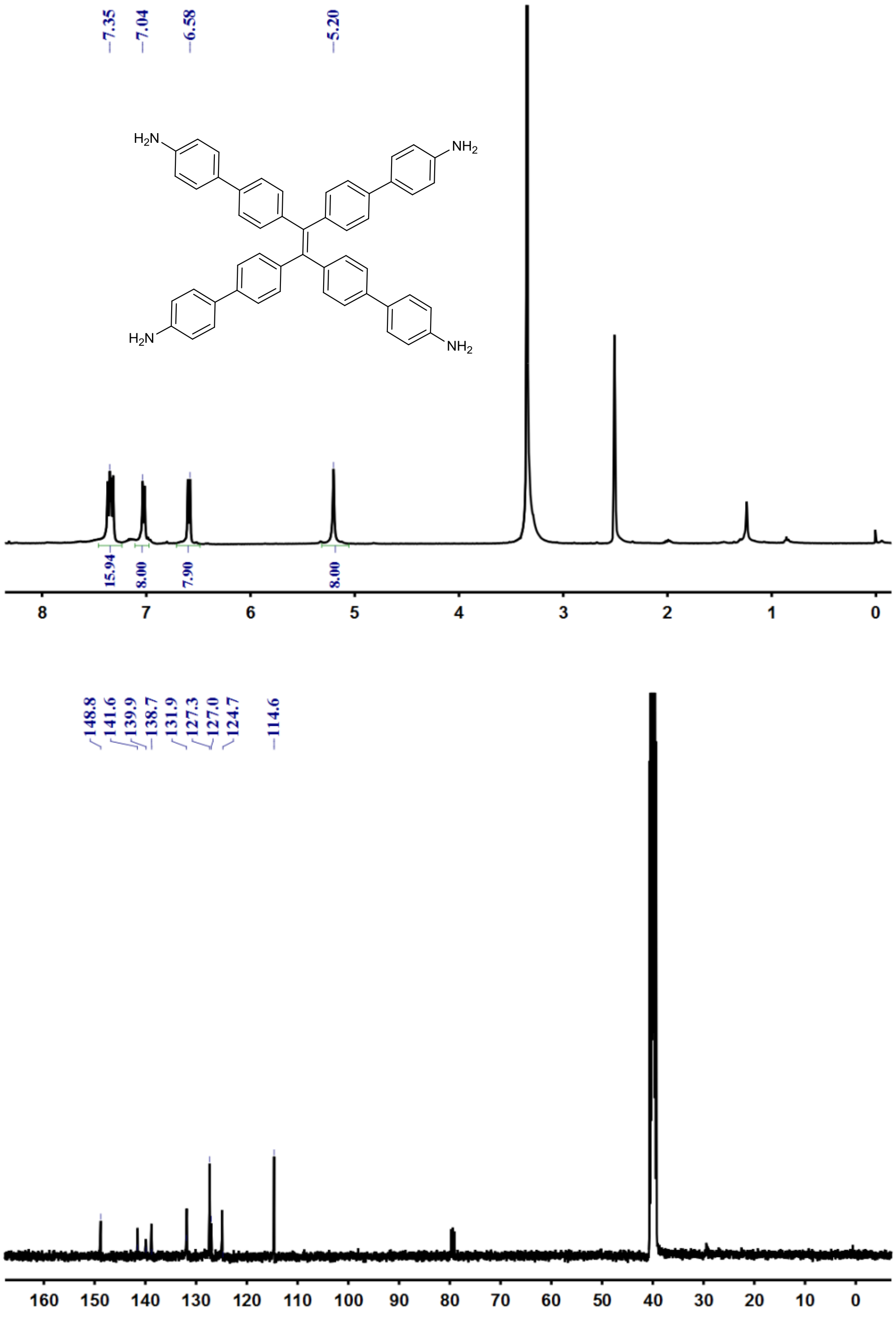

Supplementary Figure 12. ^{1}H NMR and ^{13}C NMR spectra of L_2 in DMSO-d_6.

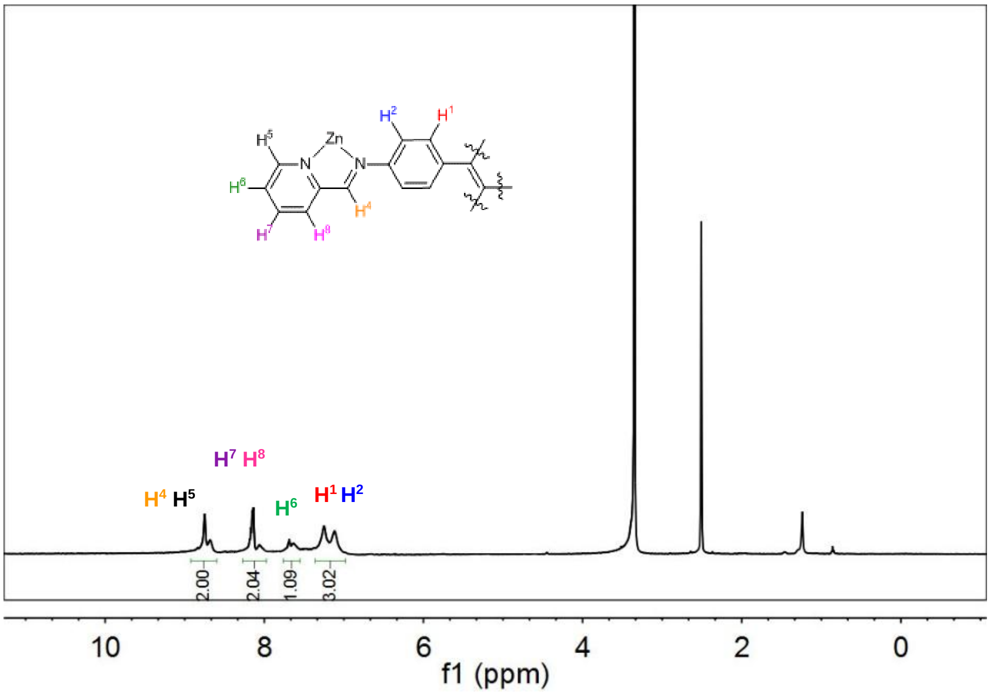

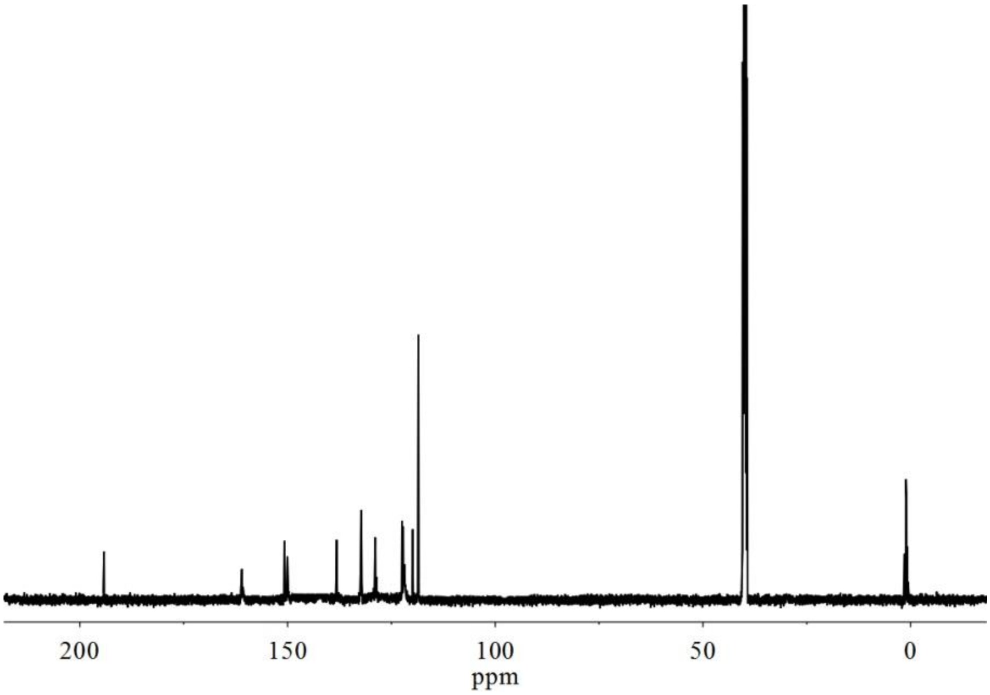

Supplementary Figure 13. ^{1}H NMR and ^{13}C NMR spectra of TPE-1 in DMSO-d_6.

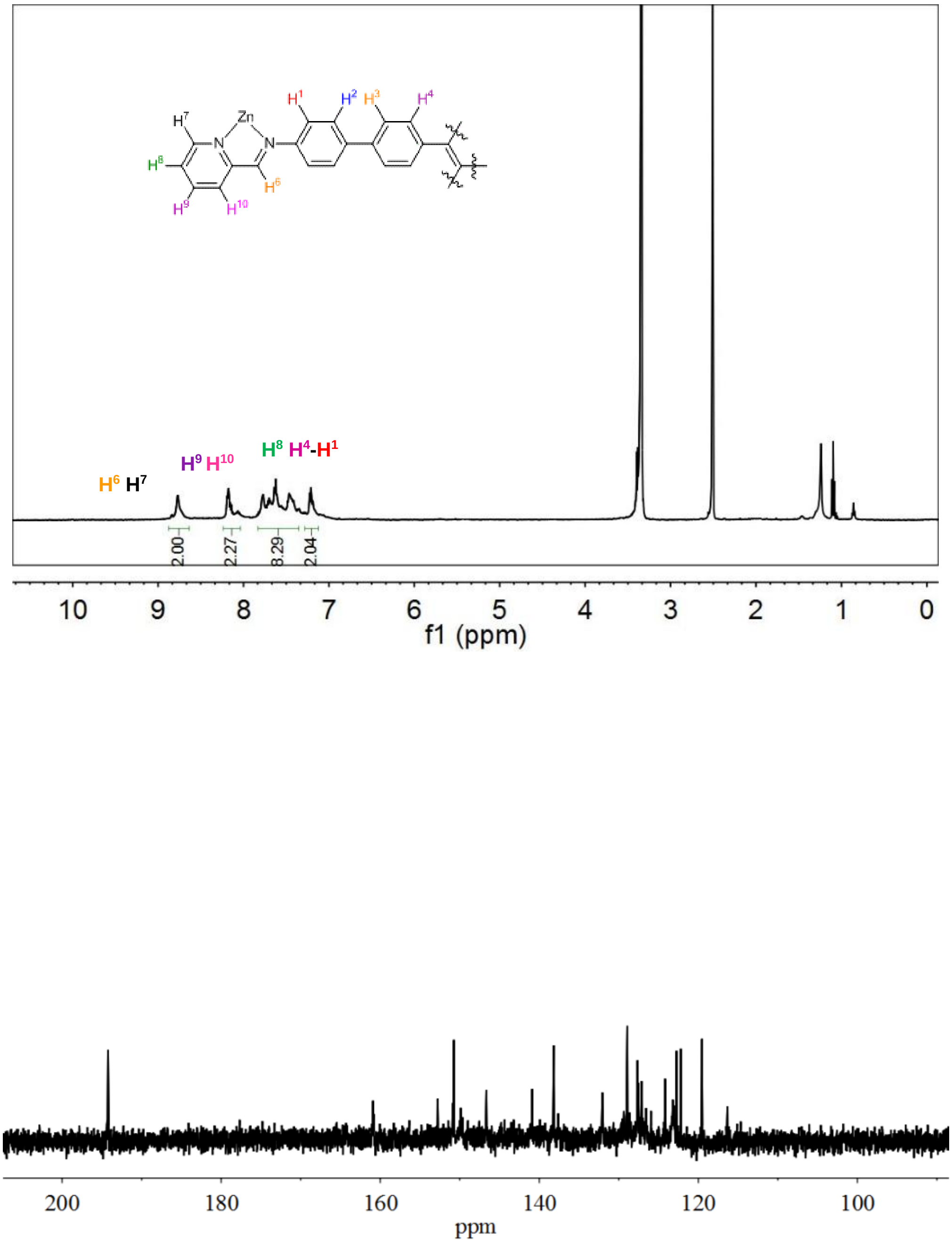

Supplementary Figure 14. ^{1}H NMR and ^{13}C NMR spectra of TPE-2 in DMSO-d_6.

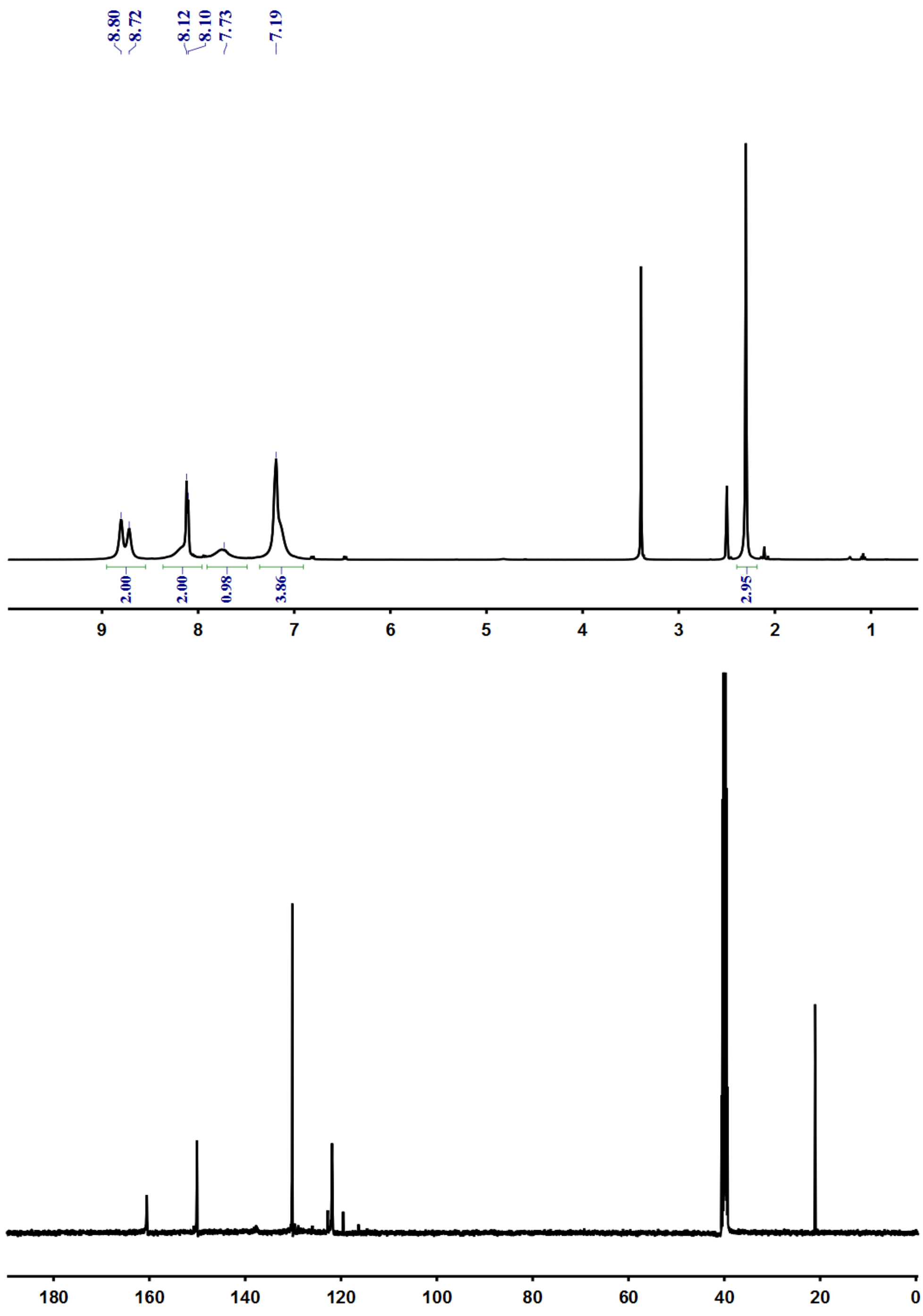

Supplementary Figure 15. **^{1}H NMR and ^{13}C NMR spectra of Zn(PI)₃ in DMSO-*d*₆.**

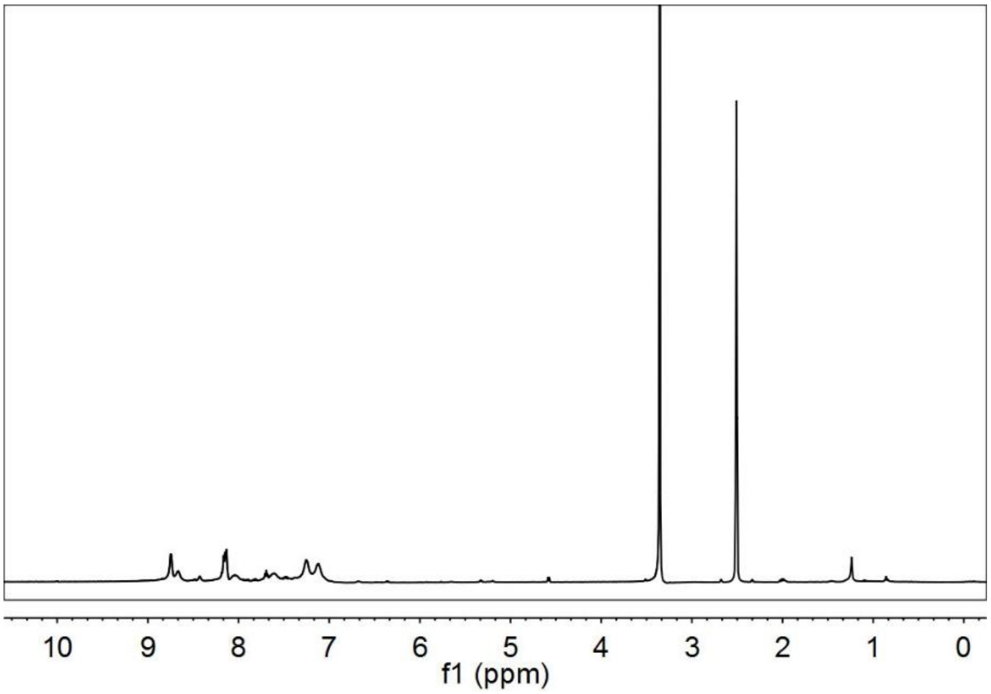

Supplementary Figure 16. ^{1}H NMR spectrum of TPE-1 after catalysis.

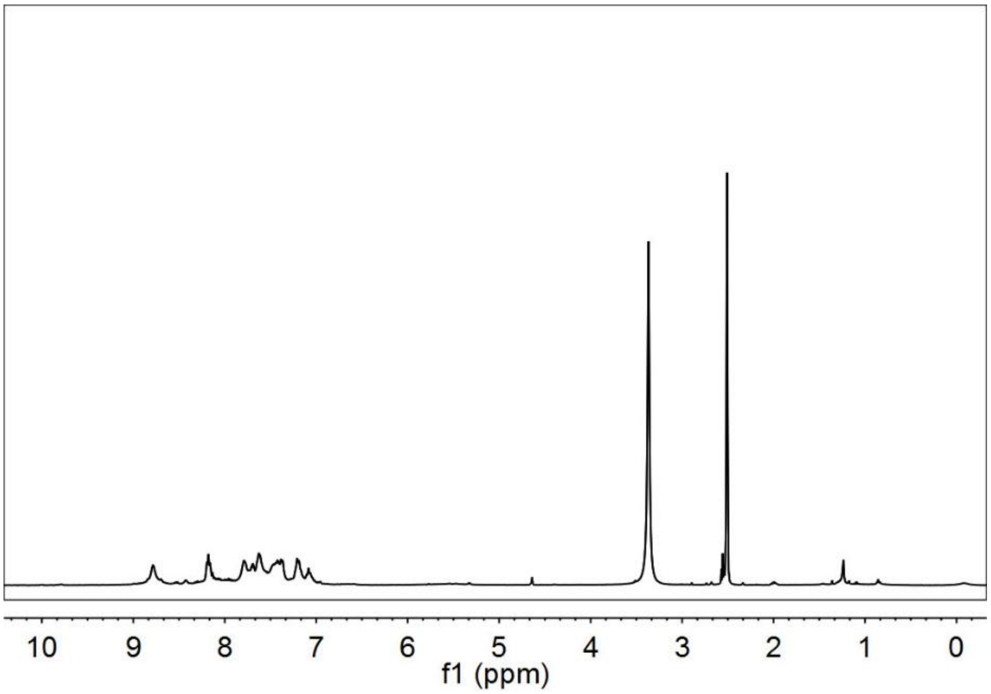

Supplementary Figure 17. ^{1}H NMR spectrum of TPE-2 after catalysis.

a

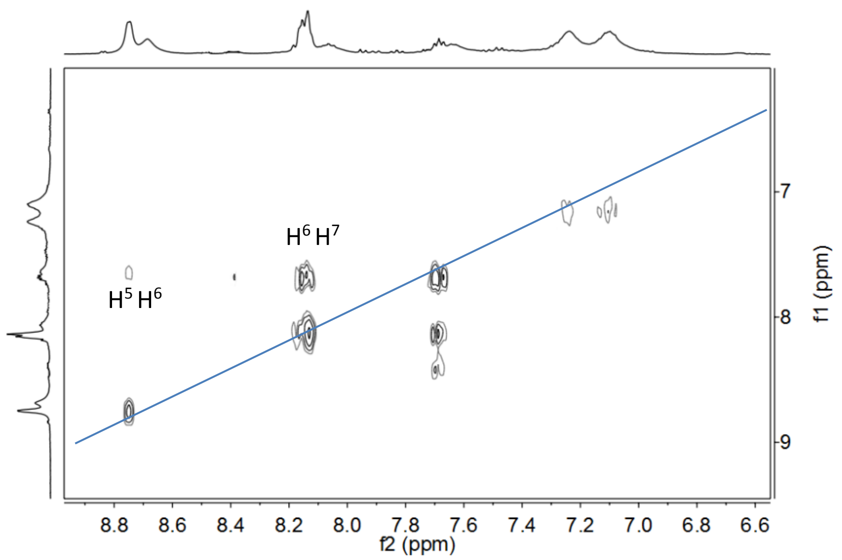

b

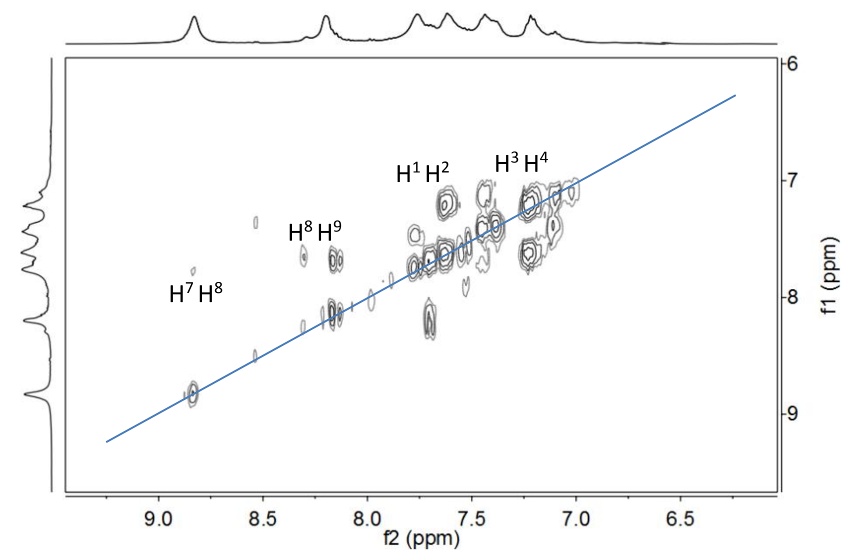

Supplementary Figure 18. ^{1}H-^{1}H COSY NMR specta of (a) TPE-1 and (b) TPE-2 in DMSO-d_6.

a

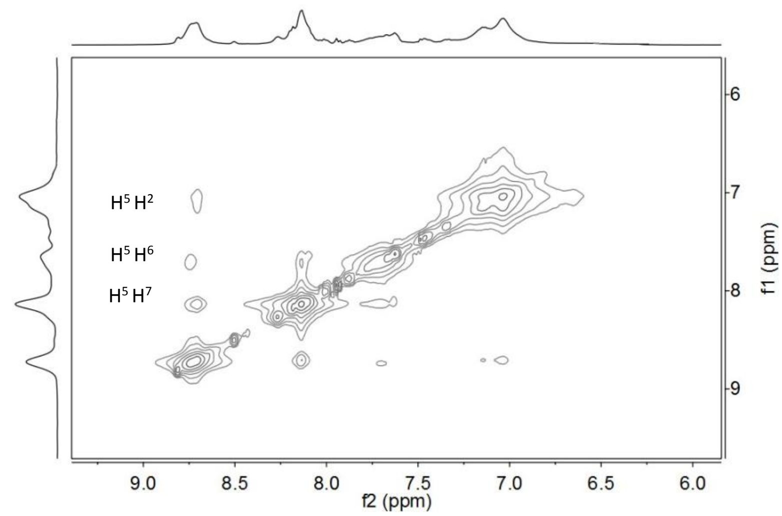

b

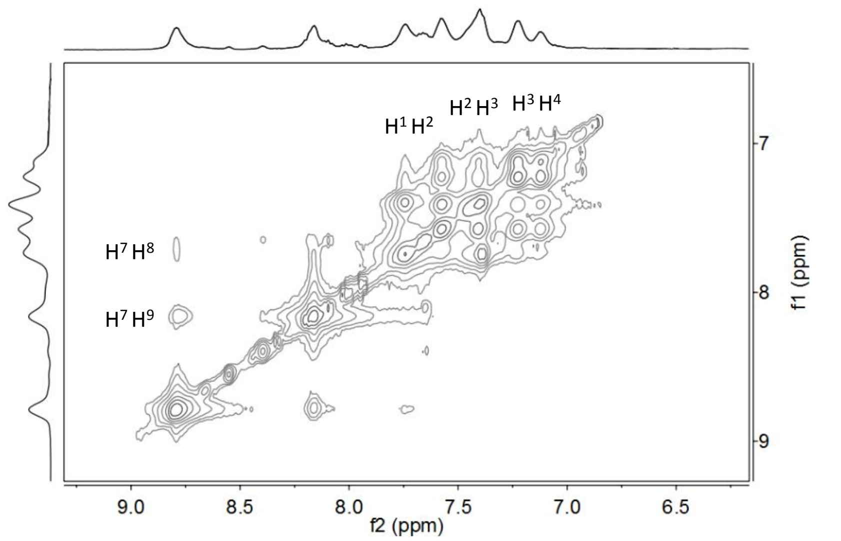

Supplementary Figure 19. NOESY NMR spectra of (a) TPE-1 and (b) TPE-2 in DMSO-d_6.

a

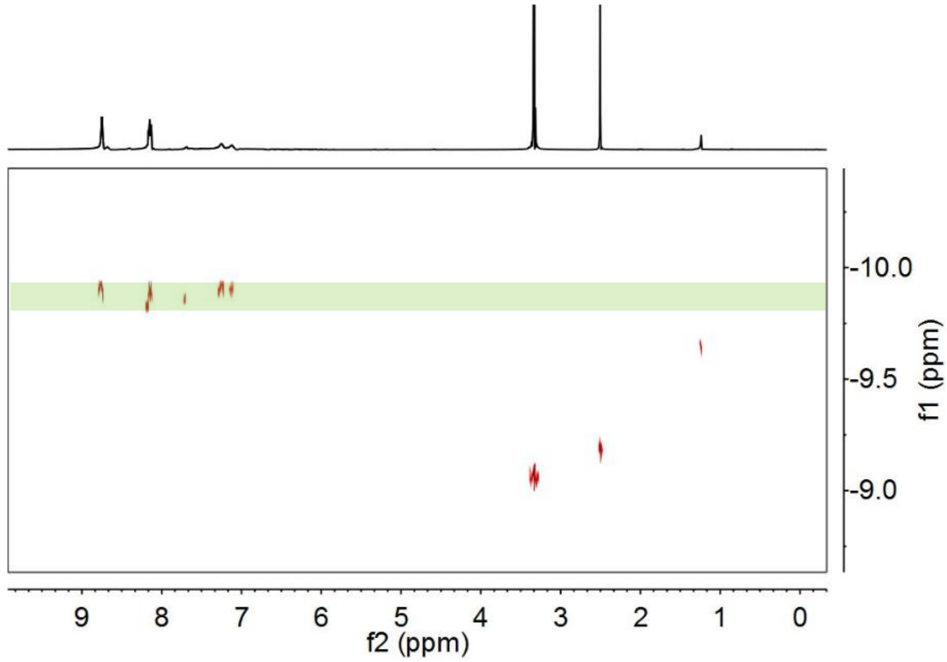

b

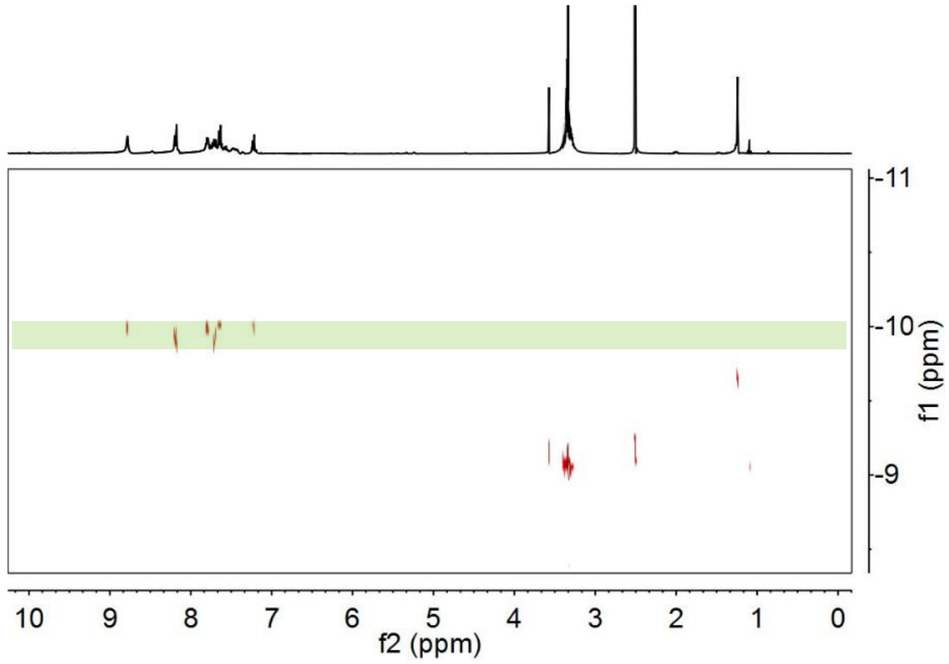

Supplementary Figure 20. ^{1}H-DOSY NMR spectra of (a) TPE-1 and (b) TPE-2 in DMSO-d_6.

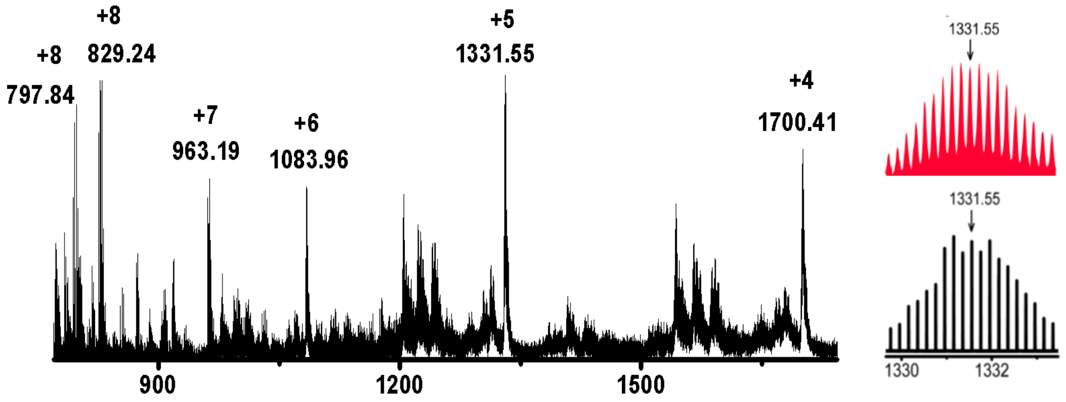

Fragment	m/z
$[Zn_8(\mathbf{L}_1)_6 \cdot 8OTf \cdot THF \cdot 2CH_3CN]^{8+}$	797.84
$[Zn_8(\mathbf{L}_1)_6 \cdot 8OTf \cdot 2THF \cdot 6CH_3CN \cdot H_2O]^{8+}$	829.24
$[Zn_8(\mathbf{L}_1)_6 \cdot 9OTf \cdot 4THF \cdot CH_3CN \cdot 2H_2O]^{7+}$	963.19
$[Zn_8(\mathbf{L}_1)_6 \cdot 10OTf]^{6+}$	1083.96
$[Zn_8(\mathbf{L}_1)_6 \cdot 11OTf]^{5+}$	1331.55
$[Zn_8(\mathbf{L}_1)_6 \cdot 12OTf]^{4+}$	1700.41

Supplementary Figure 21. Q-TOF-MS spectra of TPE-1.

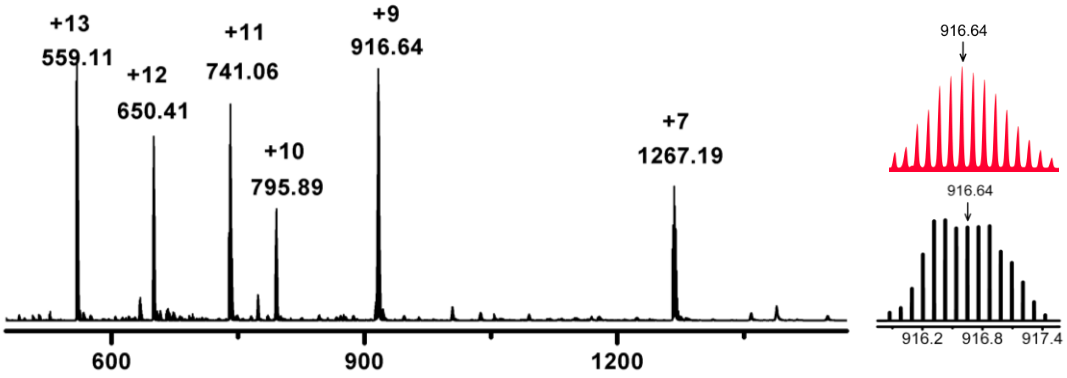

Fragment	m/z
$[Zn_8(\mathbf{L}_2)_6 \cdot 3OTf]^{13+}$	559.11
$[Zn_8(\mathbf{L}_2)_6 \cdot 4OTf \cdot 4(1,4\text{-dioxane}) \cdot THF \cdot CH_3CN]^{12+}$	650.41
$[Zn_8(\mathbf{L}_2)_6 \cdot 5OTf \cdot 5(1,4\text{-dioxane}) \cdot THF \cdot CH_3CN]^{11+}$	741.06
$[Zn_8(\mathbf{L}_2)_6 \cdot 6OTf \cdot 2(1,4\text{-dioxane}) \cdot CH_3CN]^{10+}$	795.89
$[Zn_8(\mathbf{L}_2)_6 \cdot 7OTf \cdot 3(1,4\text{-dioxane}) \cdot THF \cdot CH_3CN]^{9+}$	916.64
$[Zn_8(\mathbf{L}_2)_6 \cdot 9OTf \cdot 7(1,4\text{-dioxane}) \cdot THF]^{7+}$	1267.19

Supplementary Figure 22. Q-TOF-MS spectra of TPE-2.

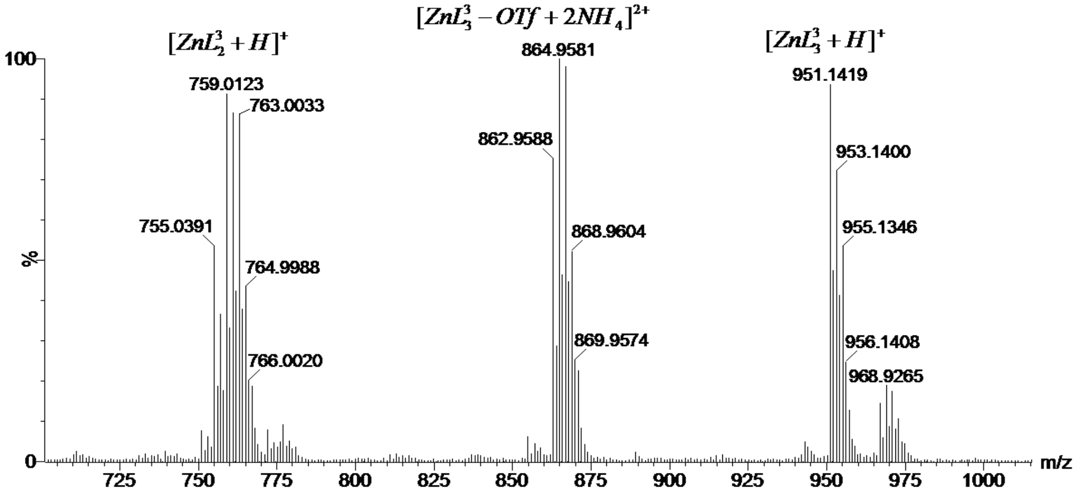

Fragment	m/z
$[Zn(PI)_2 \cdot 2OTf \cdot H]^+$	759.01
$[Zn(PI)_3 \cdot OTf \cdot CH_3CN \cdot H_2O]^+$	864.96
$[Zn(PI)_3 \cdot 2OTf \cdot H]^+$	951.14

Supplementary Figure 23. Q-TOF-MS spectra of $Zn(PI)_3$.

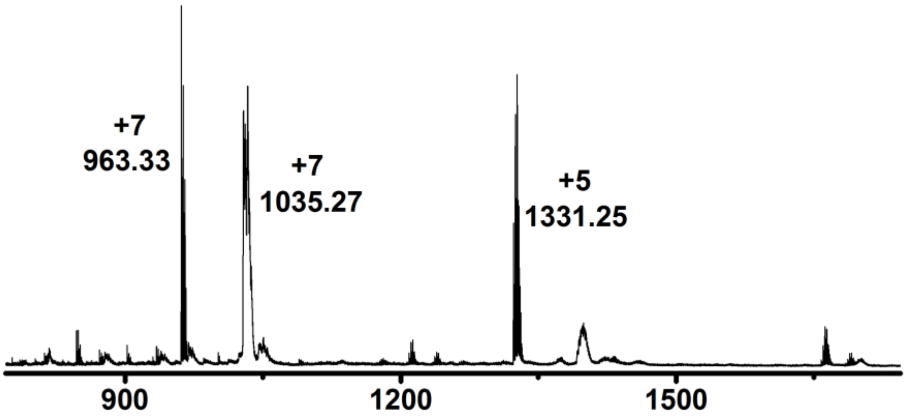

Fragment	m/z
$[Zn_8(\mathbf{L}_1)_6\cdot 9OTf\cdot 4THF\cdot CH_3CN\cdot 2H_2O]^{7+}$	963.33
$[Zn_8(\mathbf{L}_1)_6\cdot 9OTf\cdot 9THF\cdot 5CH_3CN\cdot 3H_2O]^{7+}$	1035.27
$[Zn_8(\mathbf{L}_1)_6\cdot 11OTf]^{5+}$	1331.25

Supplementary Figure 24. Q-TOF-MS spectra of TPE-1 after catalysis.

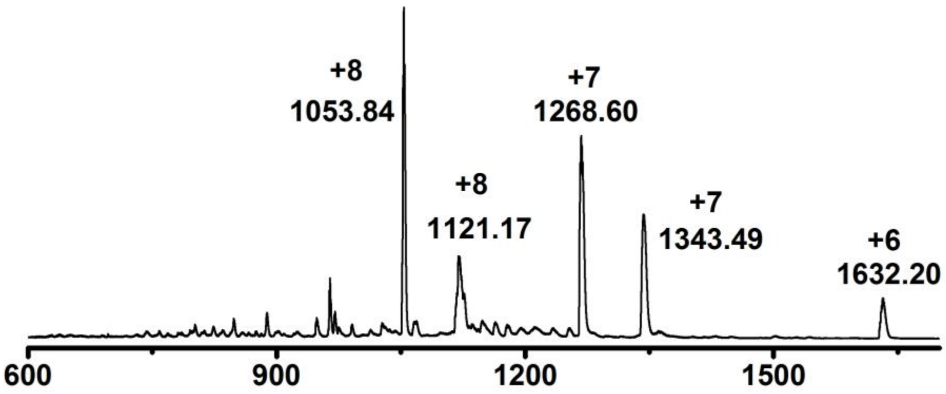

Fragment	m/z
$[Zn_8(\mathbf{L}_2)_6 \cdot 8OTf \cdot (1,4\text{-dioxane}) \cdot THF]^{8+}$	1053.84
$[Zn_8(\mathbf{L}_2)_6 \cdot 8OTf \cdot 3(1,4\text{-dioxane}) \cdot 6THF]^{8+}$	1121.17
$[Zn_8(\mathbf{L}_2)_6 \cdot 9OTf \cdot 3(1,4\text{-dioxane}) \cdot 3THF]^{7+}$	1268.60
$[Zn_8\ \mathbf{L}_2)_6 \cdot 9OTf \cdot 6(1,4\text{-dioxane})\ 6THF \cdot CH_3CN]^{7+}$	1343.49
$[Zn_8(\mathbf{L}_2)_6 \cdot 10OTf \cdot 9(1,4\text{-dioxane}) \cdot 6THF]^{6+}$	1632.20

Supplementary Figure 25. Q-TOF-MS spectra of TPE-2 after catalysis.

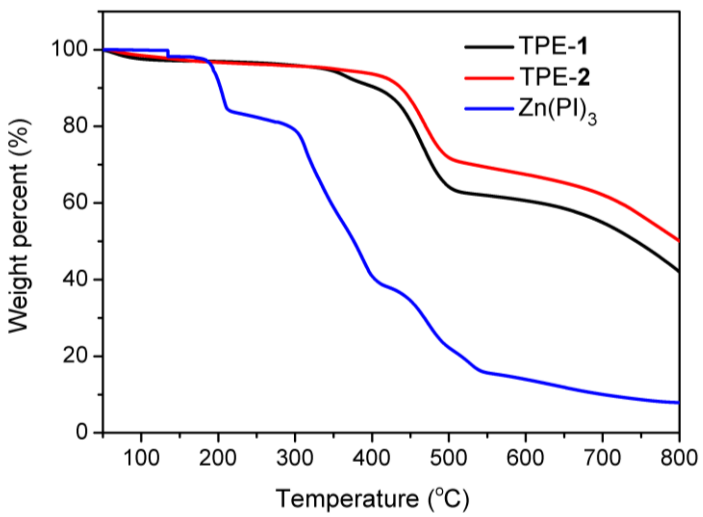

Supplementary Figure 26. TGA curves.

a

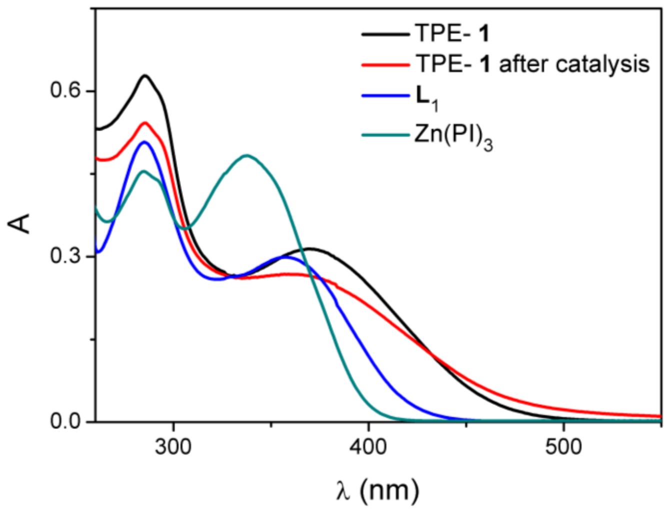

b

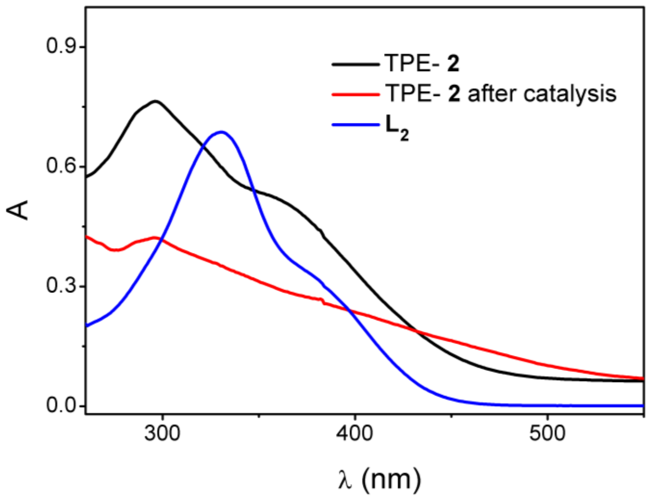

Supplementary Figure 27. UV-vis spectra of (a) L_1, TPE-1 and $Zn(PI)_3$ and (b) L_2 and TPE-2.

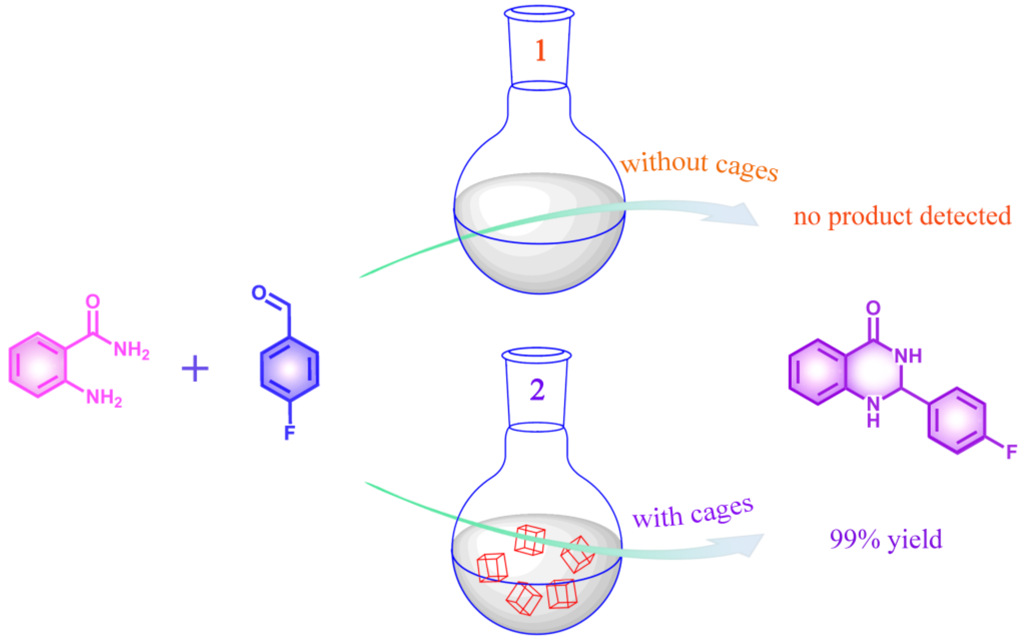

Supplementary Figure 28. Cartoon figure for the catalysis without and with cages.

Supplementary Table 8. Optimization of the Reaction solvent [a].

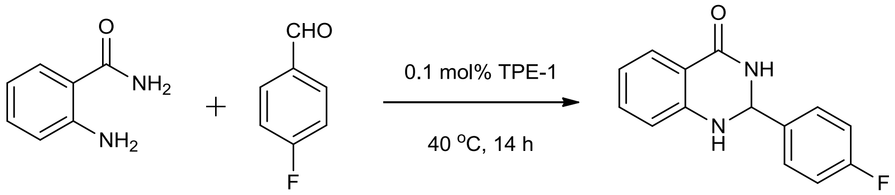

entry	solvent	yield (%)[b]
1	CH_3CN	10
2	CH_3CN : toluene = 1:2	95
3	CH_3CN : toluene = 2:1	25
4	CH_3CN: $CHCl_3$ = 1:2	36
5	CH_3CN: CH_2Cl_2 = 1:2	15
6	DMF : toluene = 2:1	8
7	CH_3CN: EtOH = 1:2	<5
8	CH_3CN: THF = 1:2	<5

[a]General conditions: catalyst TPE-**1** (0.1 mol %), **3a** (0.05 mmol), **4b** (0.055 mmol), solvent (3.0 mL), 40 °C, 1 h. [b]Isolated yield.

a

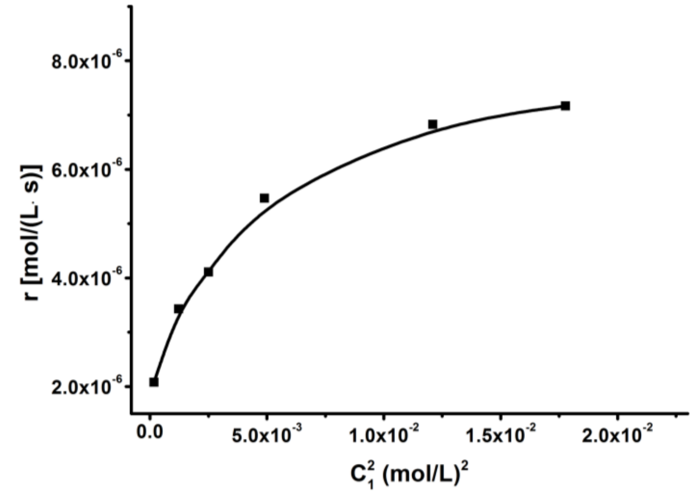

b

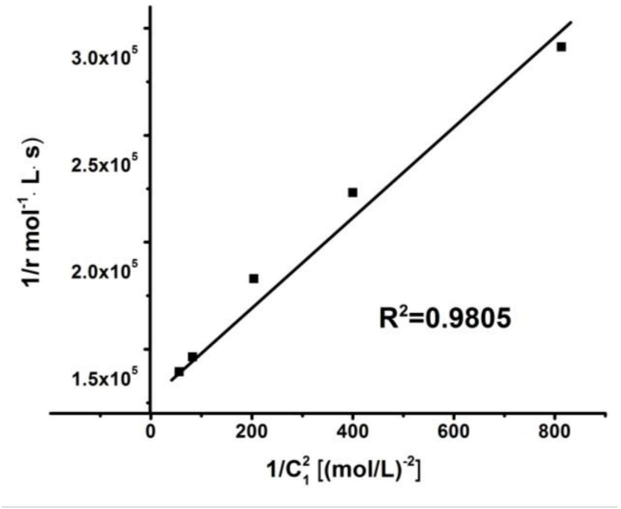

Supplementary Figure 29. (a) Steady-state kinetic analyses using the Michaelis-Menten model and (b) Lineweaver-Burk plot for TPE-1 (3a and 4b as the substrates).

a

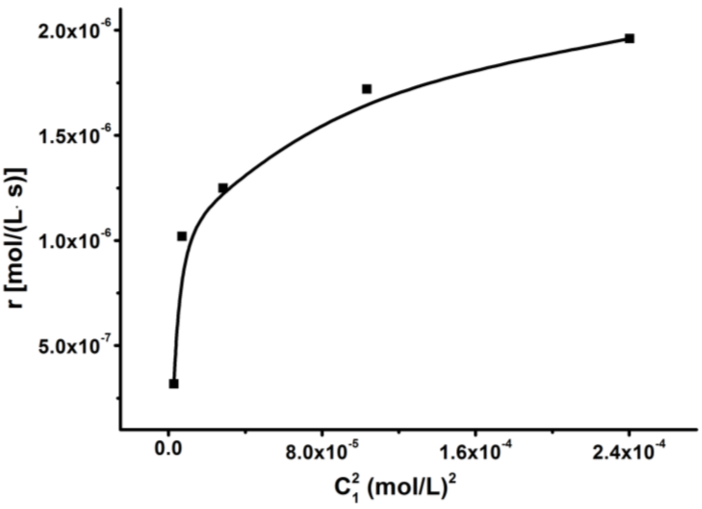

b

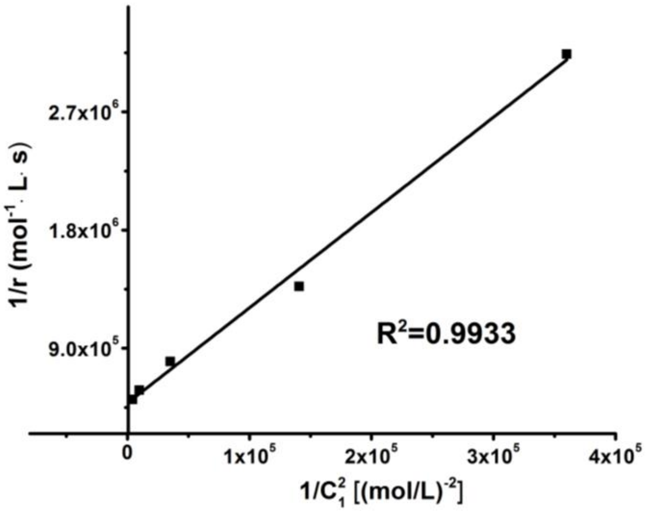

Supplementary Figure 30. (a) Steady-state kinetic analyses using the Michaelis-Menten model and (b) Lineweaver-Burk plot for TPE-2 (3a and 4b as the substrates).

a

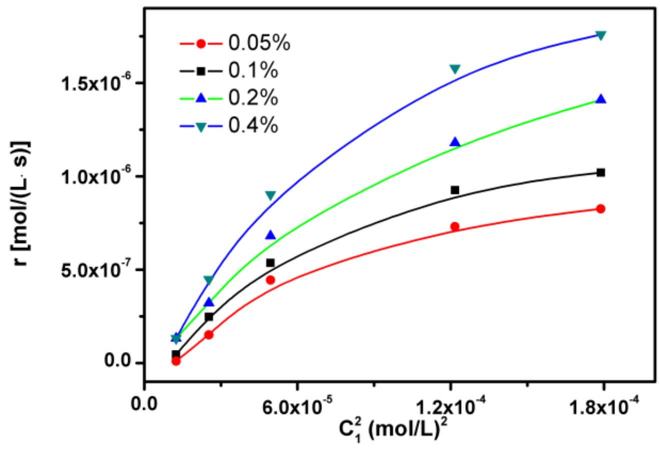

b

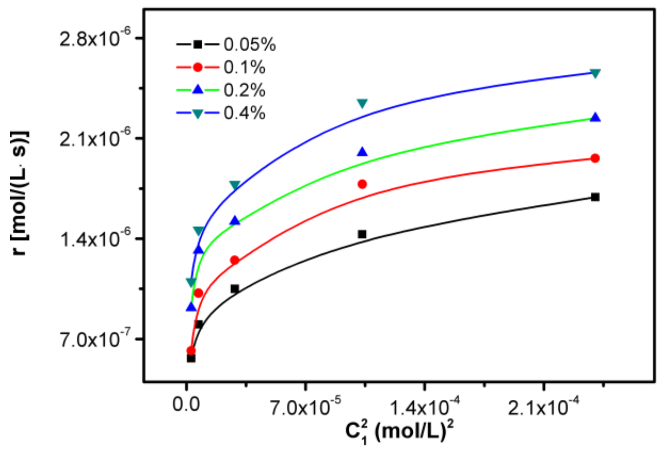

Supplementary Figure 31. Initial rate of the reaction rate as a function of the initial substrate concentration based on the Michaelis−Menten model at different loading of catalyst (a) TPE-1 and (b) TPE-2.

a

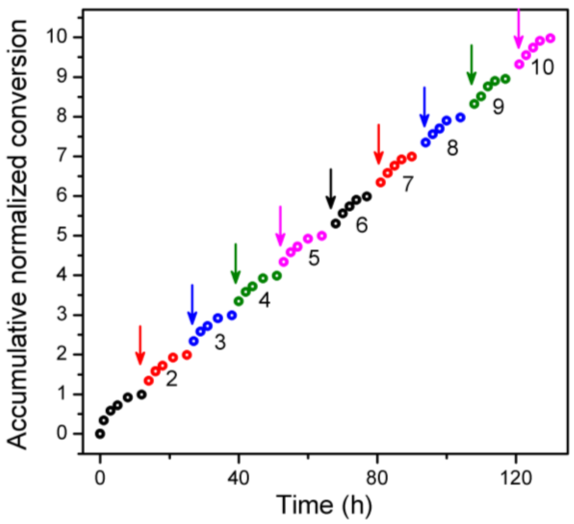

b

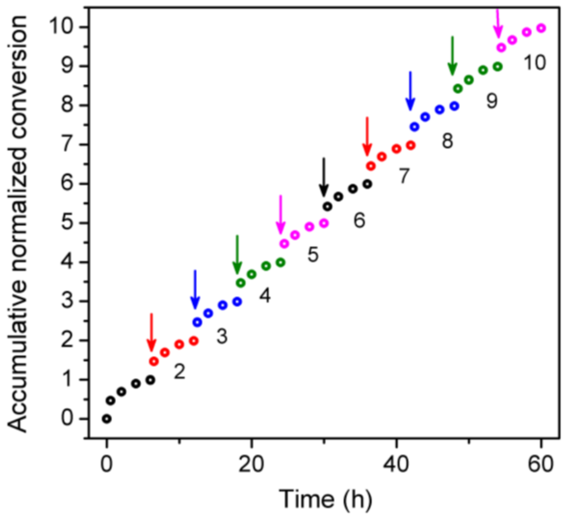

Supplementary Figure 32. Multiple turnovers catalysis results (a) TPE-1 (b) TPE-2.

a

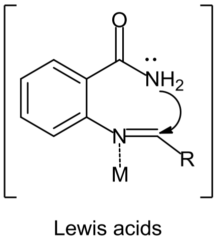

b

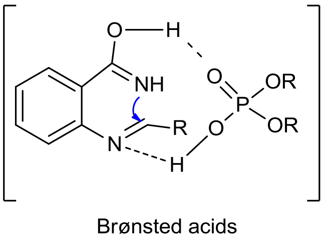

Supplementary Figure 33. Reported mechanisms for intramolecular amidation of imines catalyzed by (a) Lewis acids[13] and (b) Brønsted acids[14], respectively.

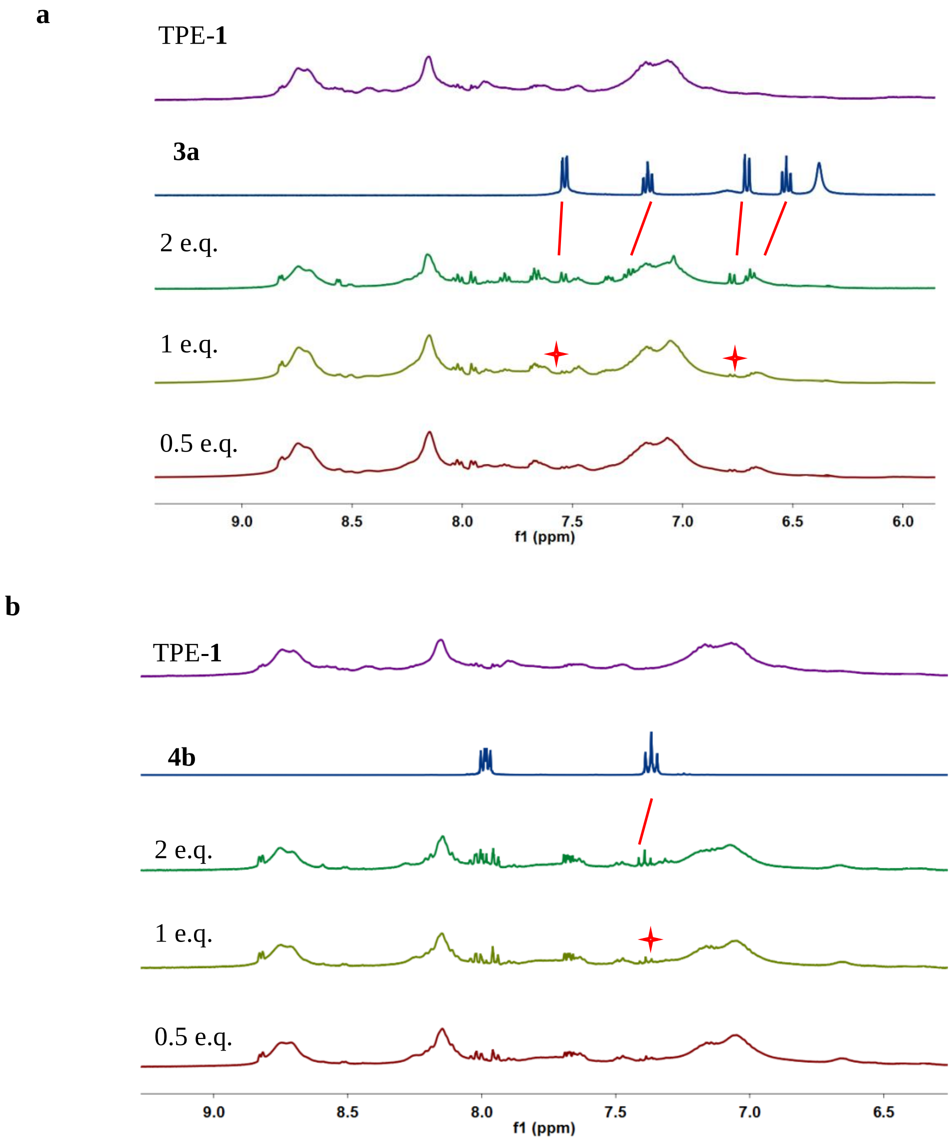

Supplementary Figure 34. ^{1}**H NMR titration of TPE-1 upon addition of substrates (a) 3a or (b) 4b.**

a

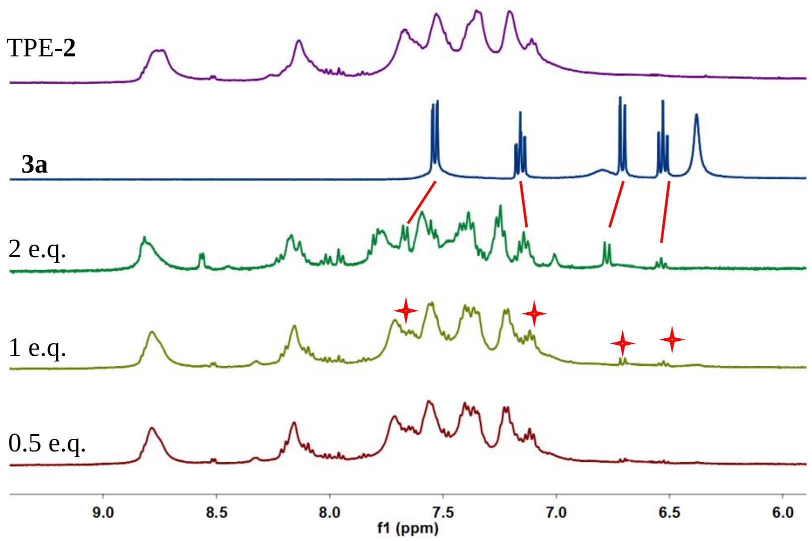

b

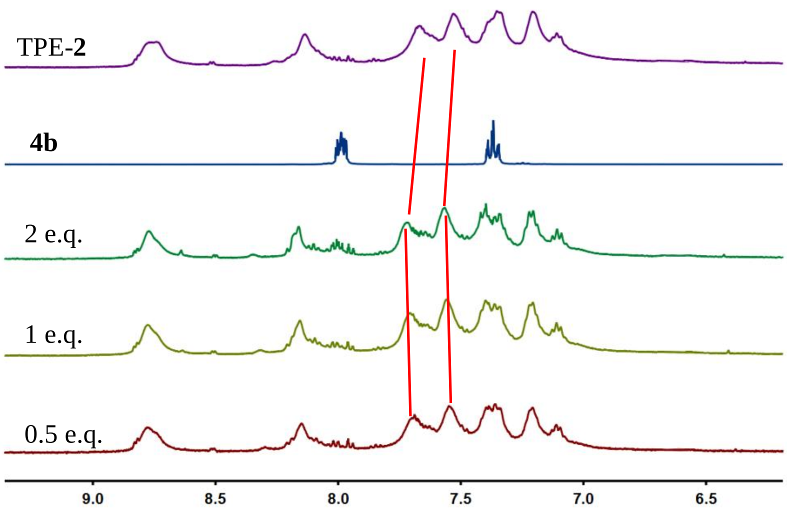

Supplementary Figure 35. **^{1}H NMR titration of TPE-2 upon addition of substrates 3a (a) or 4b (b).**

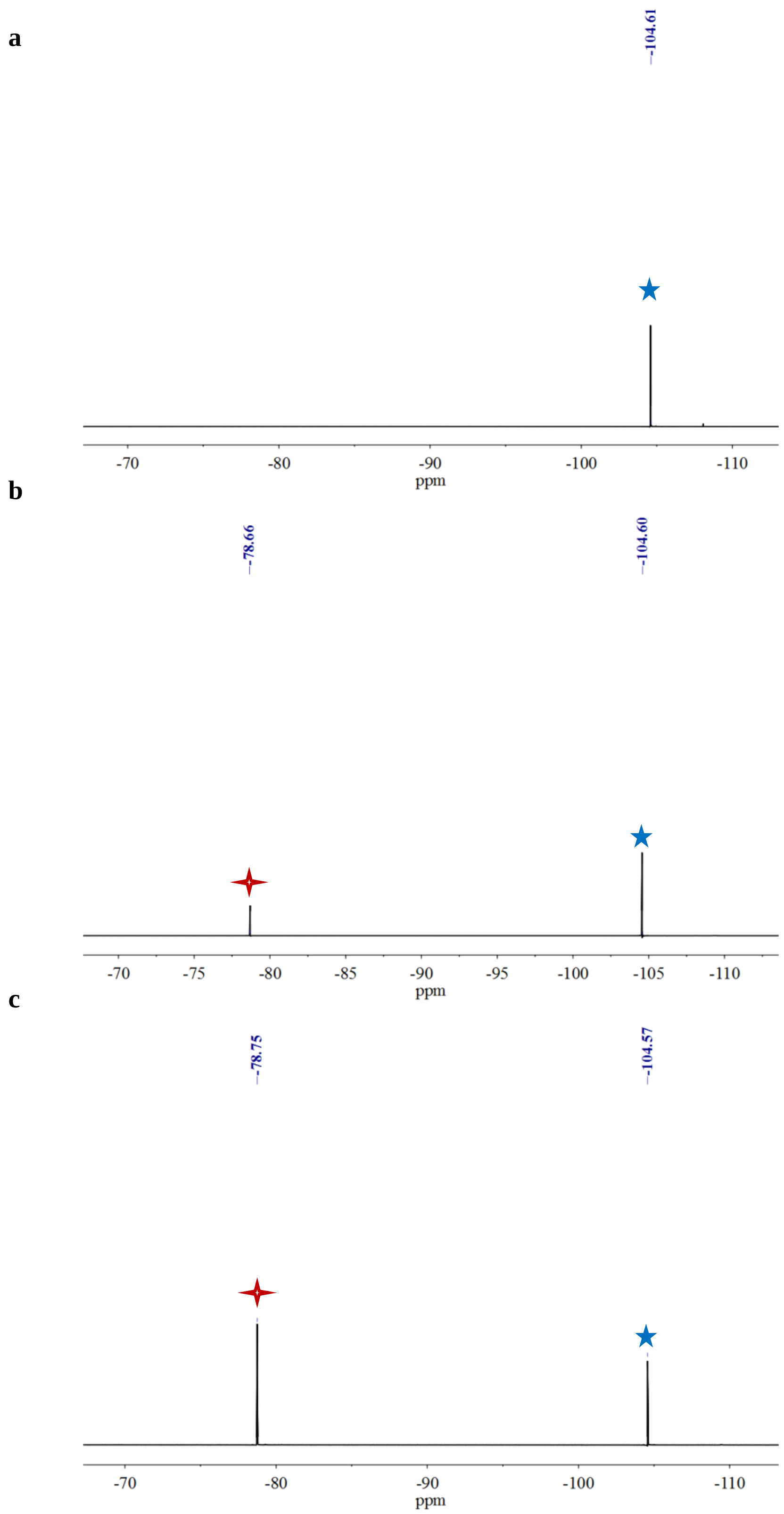

Supplementary Figure 36. ^{19}F NMR spectra of 4b (a) free state, (b) in the presence of TPE-1 (molar ratio: 4b: TPE-1 = 30:1), (c) in the presence of TPE-2 (molar ratio: 4b: TPE-2 = 30:1) in CD_3CN/DMSO-d_6 = 1:1 (v/v).

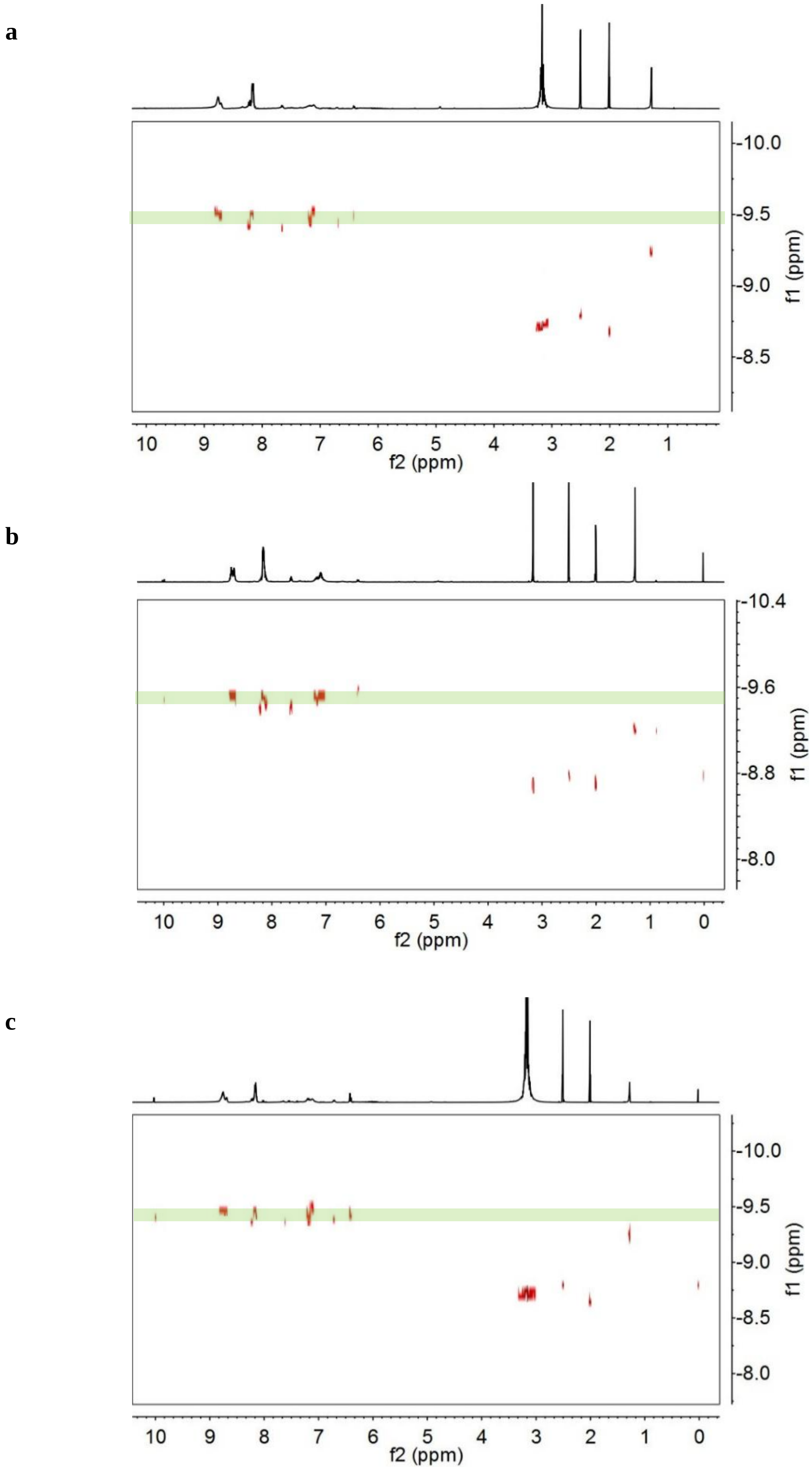

Supplementary Figure 37. **^{1}H-DOSY spectra (400 MHz) at 300 K in CD_3CN: DMSO-*d*$_6$ = 1:1 (v/v) of (a) TPE-1+3a, (b) TPE-1+4b, (c) TPE-1+3a+4b.**

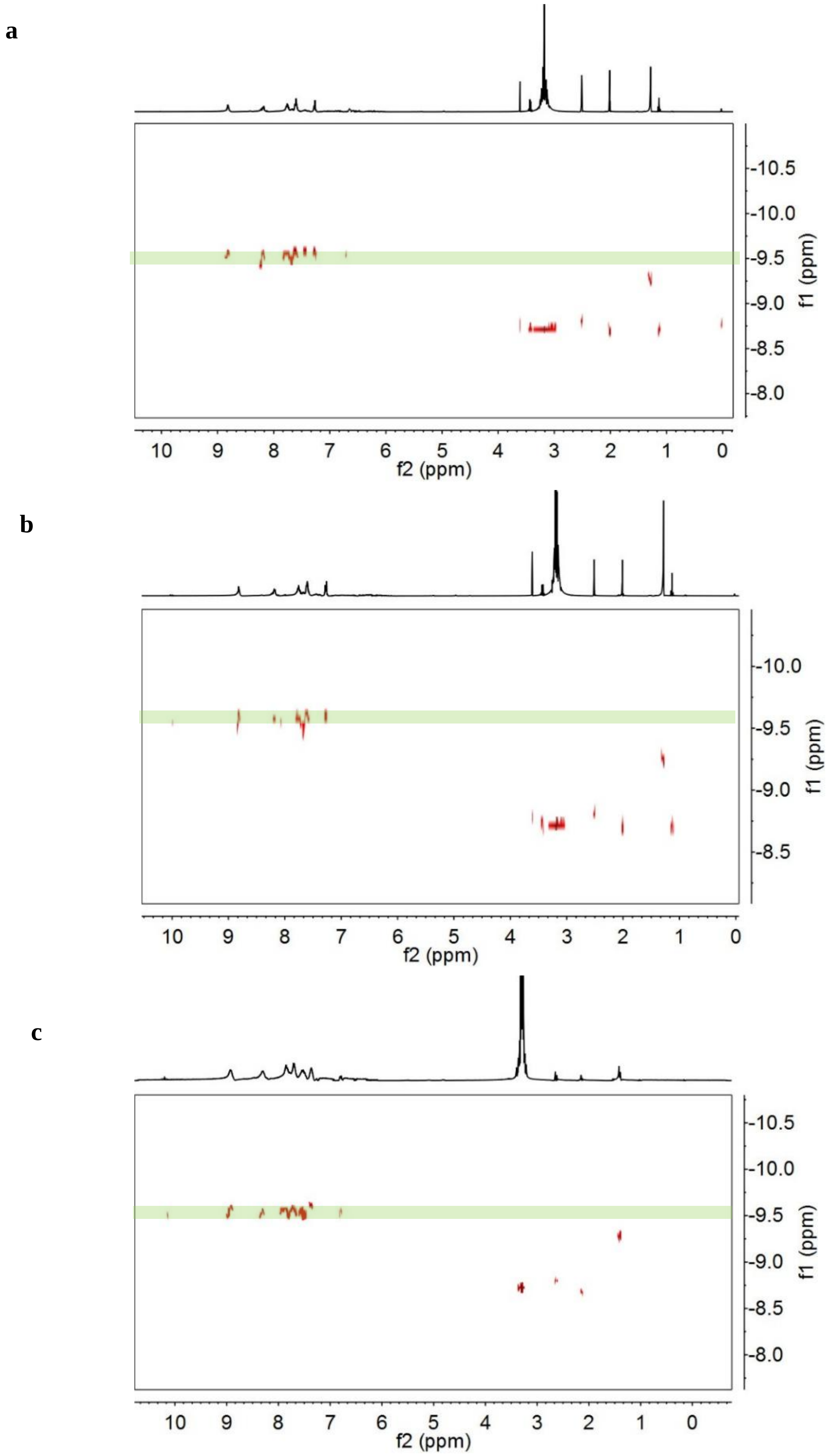

Supplementary Figure 38. **^{1}H-DOSY spectra (400 MHz) at 300 K in CD_3CN: DMSO-d_6 = 1:1 (v/v) of (a) TPE-2+3a, (b) TPE-2+4b and (c) TPE-2+3a+4b.**

a

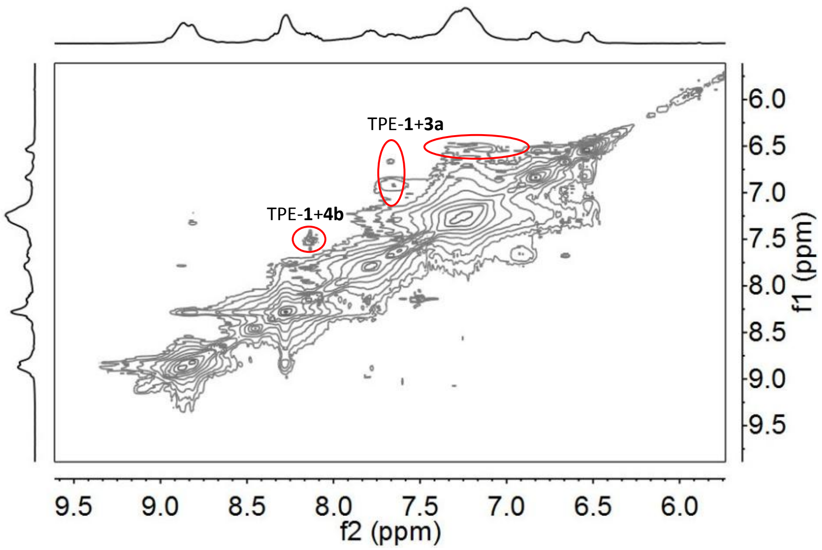

b

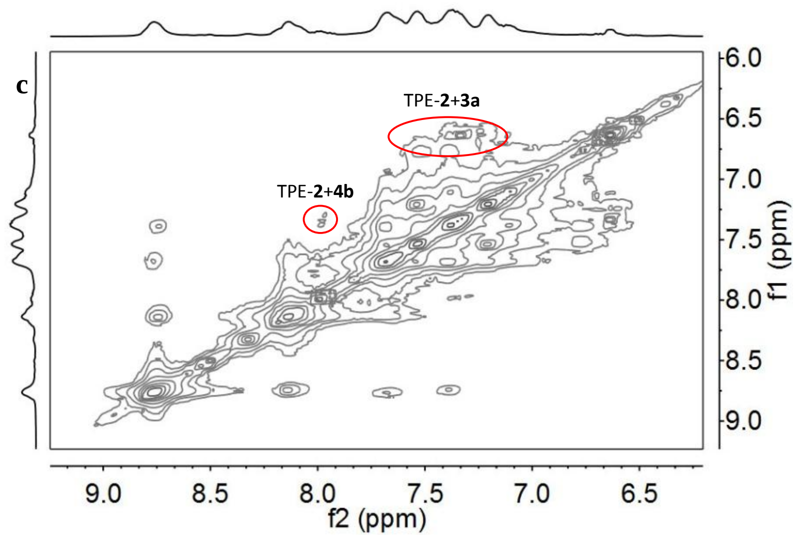

Supplementary Figure 39. NOESY spectra (400 MHz) at 300 K in CD_3CN: DMSO-d_6 = 1:1 (v/v) of (a) TPE-1+3a+4b and (b) TPE-2+3a+4b.

a

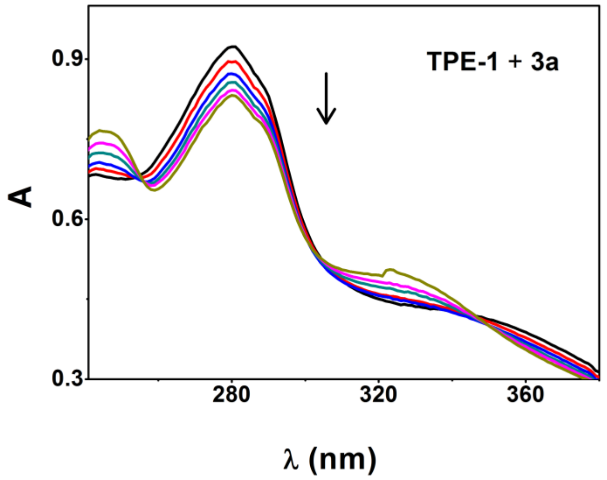

b

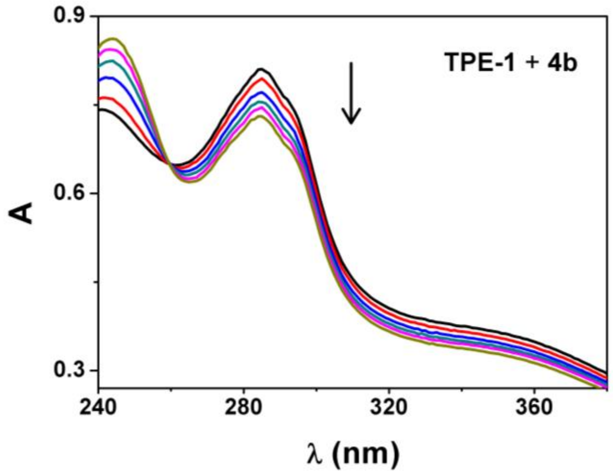

Supplementary Figure 40. UV-vis titration of the TPE-1 upon addition of (a) 3a, (b) 4b.

a

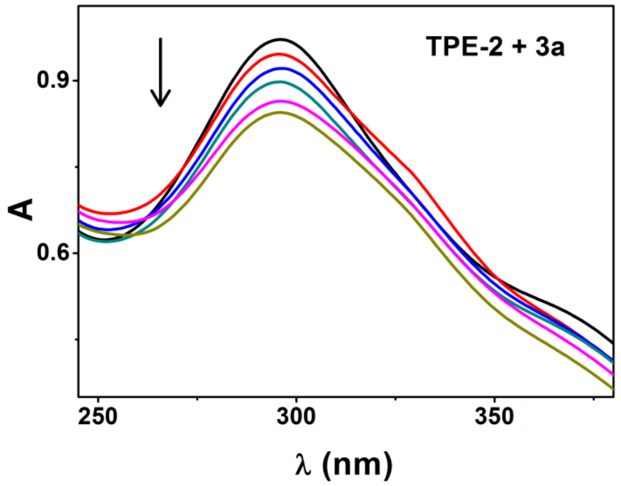

b

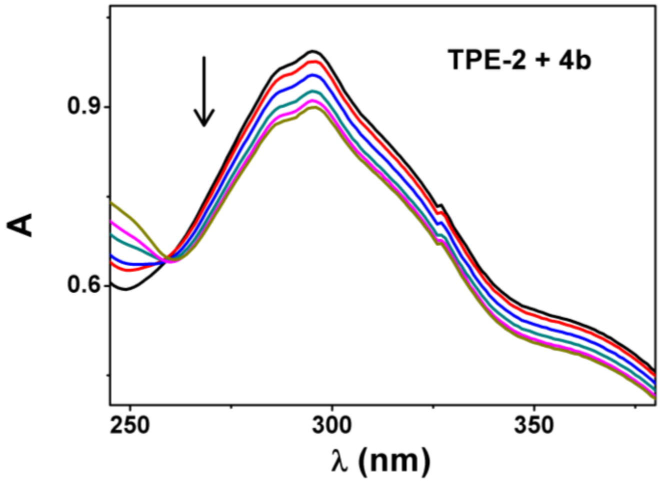

Supplementary Figure 41. UV-vis titration of the TPE-2 upon addition of (a) 3a, (b) 4b.

a b c d
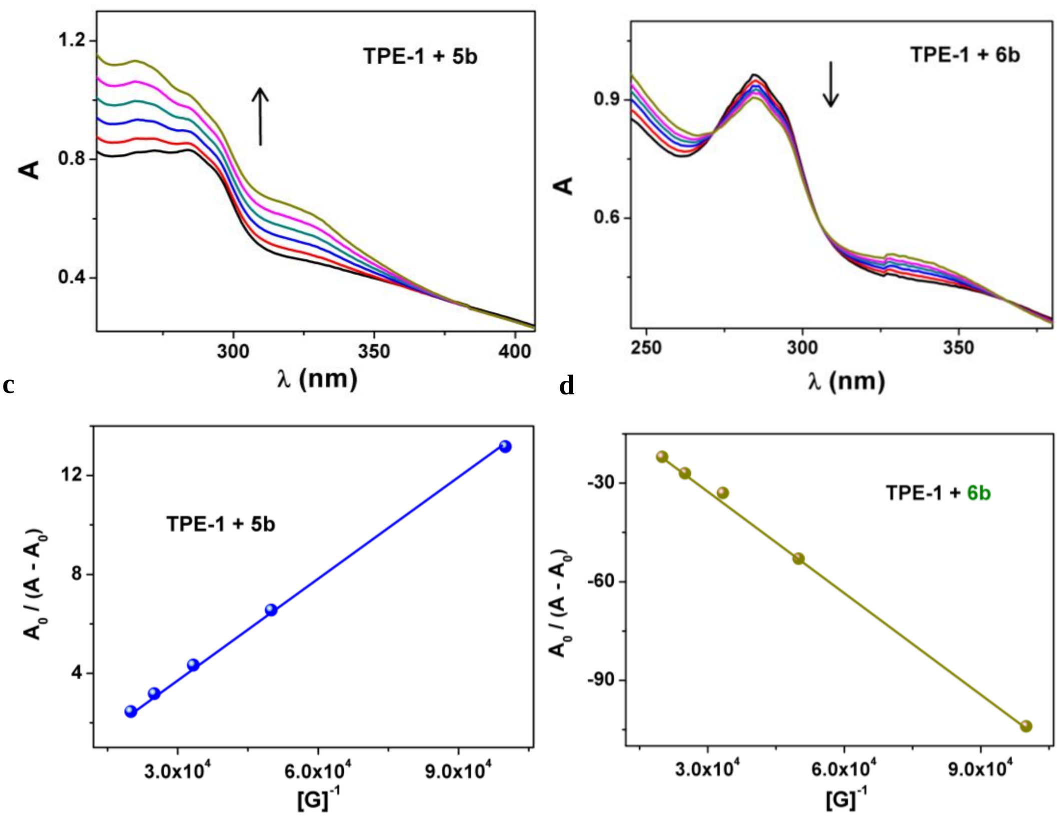

Supplementary Figure 42. UV-vis titration of the TPE-1 upon addition of (a) 5b, (b) 6b and (c, d) the corresponding B-H plots.

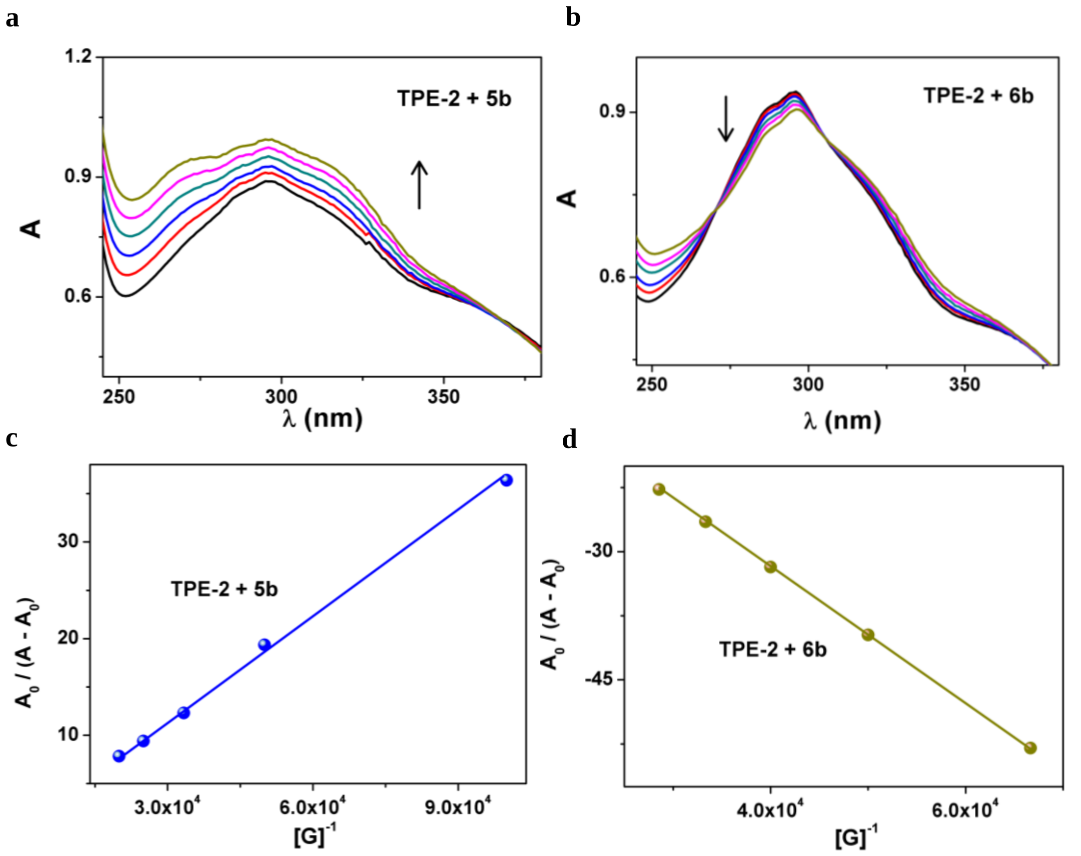

Supplementary Figure 43. UV-vis titration of the TPE-2 upon addition of (a) 5b, (b) 6b and (c, d) the corresponding B-H plots.

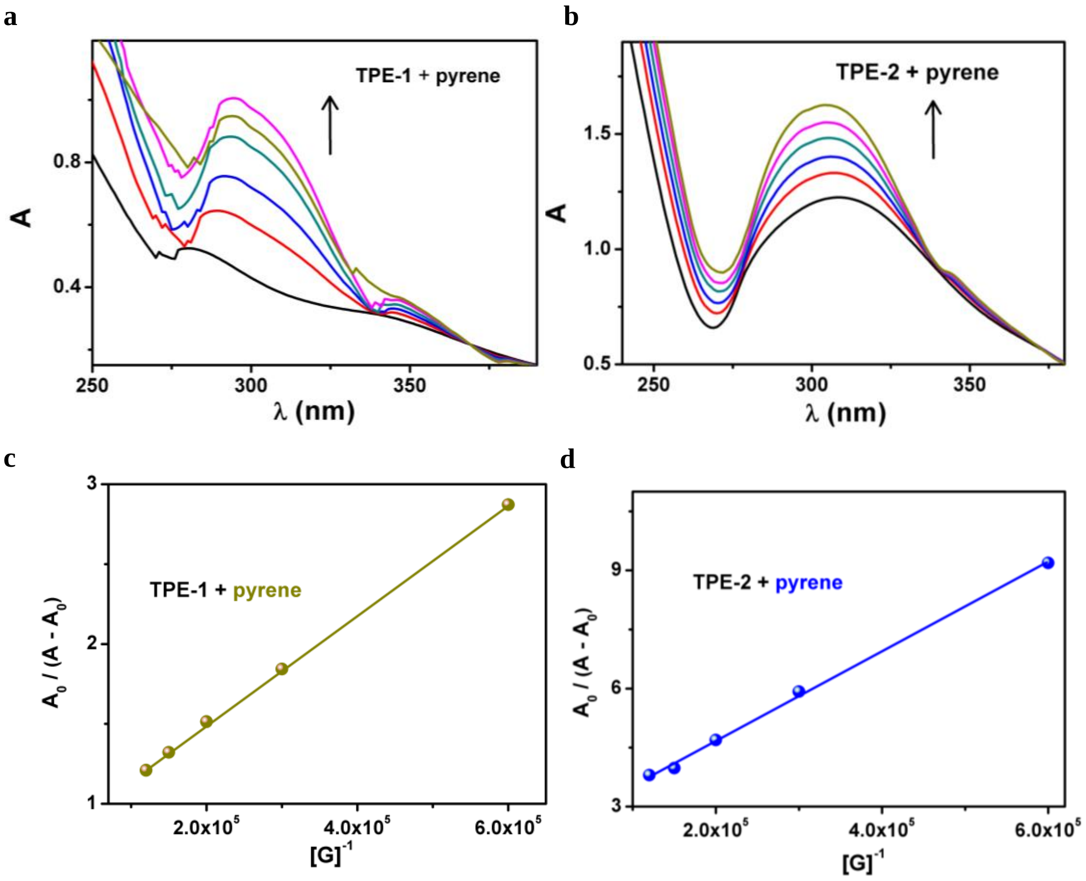

Supplementary Figure 44. UV-vis titration of the (a) TPE-1 and TPE-2 (b) upon addition of pyrene and (c, d) the corresponding B-H plots.

a

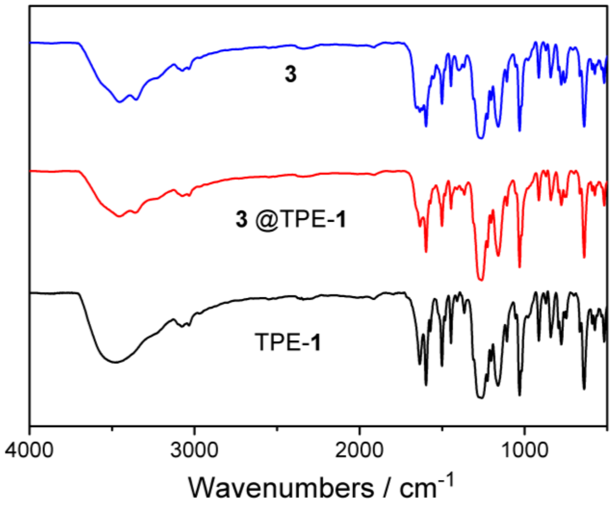

b

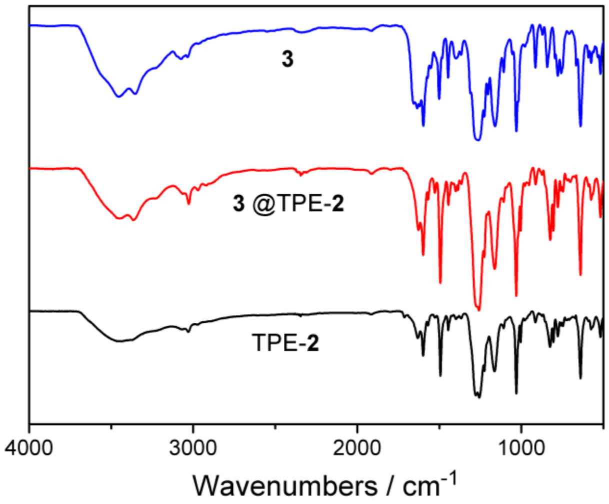

Supplementary Figure 45. IR spectra for (a) 3a@TPE-1 and (b) 3a@TPE-2.

a

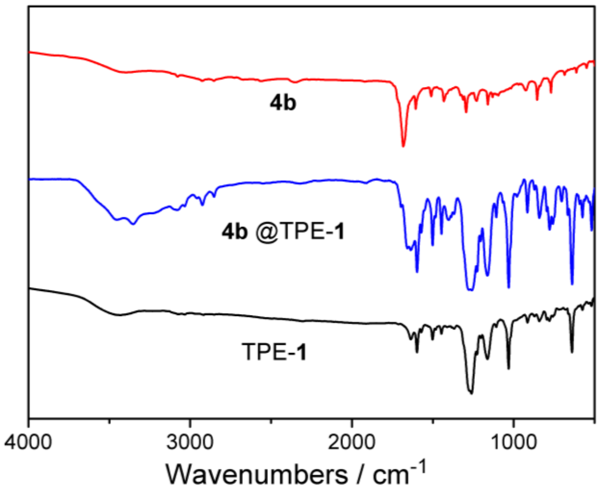

b

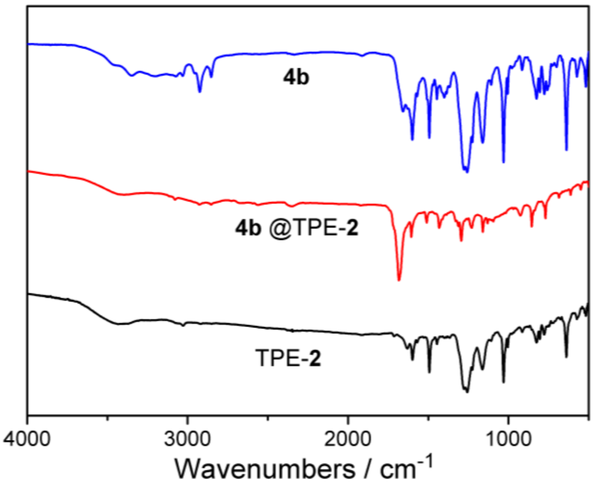

Supplementary Figure 46. IR spectra for (a) 4b@TPE-1 and (b) 4b@TPE-2.

a

b

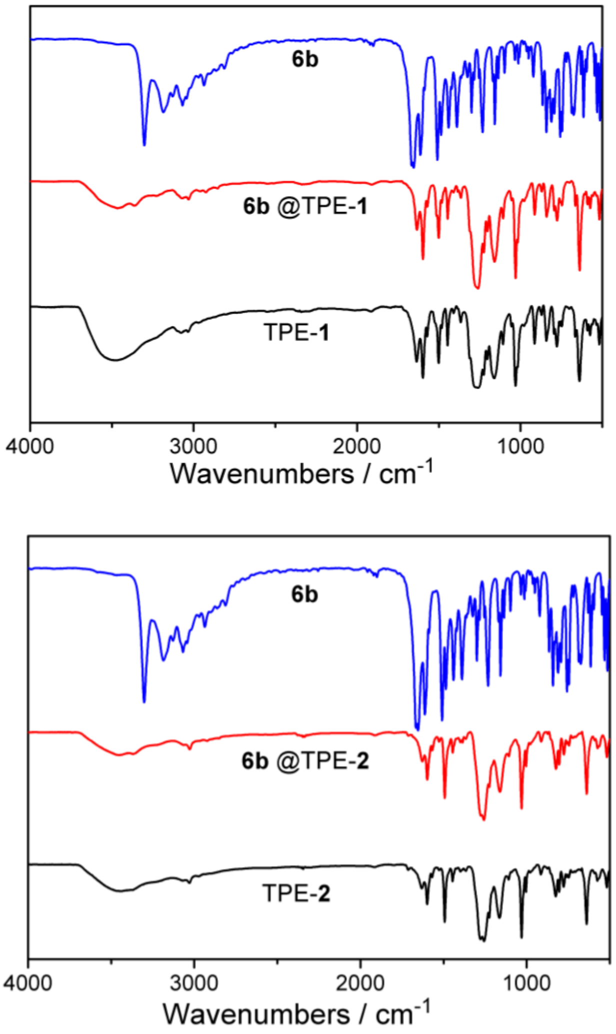

Supplementary Figure 47. IR spectra for (a) 6b@TPE-1 and (b) 6b@TPE-2.

Supplementary References.

1 Sheldrick, G. M. SHELXT-2014, (2013).

2 Dolomanov, O. V., Bourhis, L. J., Gildea, R. J., Howard, J. A. & Puschmann, K. H. OLEX2: a complete structure solution, refinement and analysis program. *J. Appl. Crystallogr.* **42** 339-341 (2009).

3 Zhang, L. L., Yu, X. Y., Zhang, L. X., Zhou, X. G. & Lin, Y. H. *Org. Chem. Front.* **1** 929-935 (2014).

4 Lu, J. & Zhang, J. Facile synthesis of azo-linked porous organic frameworks via reductive homocoupling for selective CO_2 capture *J. Mater. Chem. A* **2** 13831-13834 (2014).

5 Hastings, C. J., Pluth, M. D., Bergman, R. G. & Raymond, K. N. J. Am. Chem. Soc., 132 6938-6940 (2010).

6 Cullen, W., Misuraca, M. C., Hunter, C. A., Williams, N. H. & Ward, M. D. Nat. Chem., 8 231-236 (2016).

7 Kaphan, D. M., Levin, M. D., Bergman, R. G., Raymond, K. N. & Toste, F. D. Science 350 1235-1238 (2015).

8 Hastings, C. J., Fiedler, D., Bergman, R. G. & Raymond, K. N. J. Am. Chem. Soc., 130 10977-10983 (2008).

9 Fiedler, D., Halbeek, H., Bergman, R. G. & Raymond, K. N. J. Am. Chem. Soc., 128 10240-10252 (2006).

10 Pluth, M. D., Bergman, R. G. & Raymond, K. N. J. Am. Chem. Soc., 130 11423-11429 (2008).

11 Pluth, M. D., Bergman, R. G. & Raymond, K. N. Science 316 85-88 (2007).

12 Bolliger, J. L., Belenguer A. M. & Nitschke. J. R. Angew. Chem. Int. Ed., 52 7958-7962 (2013).

13 Prakash, M., Jayakumar, S. & Kesavan, V. Investigation of the Enantioselective Synthesis of 2,3-Dihydroquinazolinones Using Sc(III)-*inda*-pybox. *Synthesis* **45** 2265-2272 (2013).

14 Huang, D., Li, X., Xu, F., Li, L. & Lin X. Highly Enantioselective Synthesis of Dihydroquinazolinones Catalyzed by SPINOL-Phosphoric Acids. *ACS Catal.* **3** 2244-2247 (2013).
